# Widespread genomic *de novo* DNA methylation occurs following CD8^+^ T cell activation and proliferation

**DOI:** 10.1080/15592294.2024.2367385

**Published:** 2024-06-20

**Authors:** Annika R. Seddon, Olivia M. Damiano, Mark B. Hampton, Aaron J. Stevens

**Affiliations:** a Department of Pathology and Biomedical Science, Mātai Hāora - Centre for Redox Biology and Medicine, University of Otago, Christchurch, New Zealand; b Department of Pathology and Molecular Medicine, Genetics and Epigenetics Research Group, University of Otago, Wellington, New Zealand

**Keywords:** T-cell epigenetics, inflammation, redox biology, DNA methylation, oxidants, differentiation, proliferation, CD3/CD28

## Abstract

This research investigates the intricate dynamics of DNA methylation in the hours following CD8+ T cell activation, during a critical yet understudied temporal window. DNA methylation is an epigenetic modification central to regulation of gene expression and directing immune responses. Our investigation spanned 96-h post-activation and unveils a nuanced tapestry of global and site-specific methylation changes. We identified 15,626 significant differentially methylated CpGs spread across the genome, with the most significant changes occurring within the genes *ADAM10*, *ICA1*, and *LAPTM5*. While many changes had modest effect sizes, approximately 120 CpGs exhibited a log_2_FC above 1.5, with cell activation and proliferation pathways the most affected. Relatively few of the differentially methylated CpGs occurred along adjacent gene regions. The exceptions were seven differentially methylated gene regions, with the Human T cell Receptor Alpha Joining Genes demonstrating consistent methylation change over a 3kb window. We also investigated whether an inflammatory environment could alter DNA methylation during activation, with proliferating cells exposed to the oxidant glycine chloramine. No substantial differential methylation was observed in this context. The temporal perspective of early activation adds depth to the evolving field of epigenetic immunology, offering insights with implications for therapeutic innovation and expanding our understanding of epigenetic modulation in immune function.

## Introduction

Epigenetic modifications such as DNA methylation play crucial roles in regulating gene expression by influencing the accessibility of DNA to transcriptional machinery [1]. T cell activation and proliferation are modulated by epigenetic mechanisms that shape the development of the adaptive response, however, comprehensive studies elucidating the precise changes in T cell epigenetic profiles are still required [2]. DNA methylation is an epigenetic modification that involves the addition of a methyl group to the DNA, typically at the cytosine residues of CpG dinucleotides and contributes a central role in directing the functionality of immune cells [3]. Unravelling the dynamic interplay between T cell activation and DNA methylation can provide transformative potential for therapeutic interventions and in immune-related disorders, and in understanding the regulatory mechanisms shaping cellular behaviours.

During CD8+ T cell activation, the immune system responds to a pathogen or other stimuli, leading to the proliferation and differentiation of CD8+ T cells into effector cells that can eliminate infected or aberrant cells [4,5]. The activation and proliferation of CD8+ T cells is a highly regulated immune response beginning with the T cell receptor (TCR) on their surface binding to the presented antigens. The interaction between the TCR and the antigen-MHC class I complex (Major Histocompatibility Complex class I) is a key step in activation. Co-stimulatory signals are required to fully activate CD8+ T cells, and include molecules like CD28 on the T cell surface interacting with B7 molecules on antigen-presenting cells [6]. Without co-stimulation, T cells may become tolerant to the antigen. Upon antigen recognition and co-stimulation, intracellular signalling pathways are activated within the CD8+ T cell. This leads to the production of various cytokines and the upregulation of cell surface molecules involved in effector function [7]. Activated CD8+ T cells undergo clonal expansion, where a small number of antigen-specific T cells rapidly proliferate into a large population of effector cells. This amplification is crucial for building an army of T cells capable of responding to the infection [8]. During clonal expansion, CD8+ T cells differentiate into effector cells with specific functions. This includes cytotoxic T cells, which directly kill infected cells, and memory T cells, which persist in circulation poised to proliferate, providing long-term protection against the encountered pathogen [9]. Epigenetic regulation, including DNA methylation, is known to be involved in CD8+ T cell differentiation [10], response to tumours [11] and exhaustion [12]; however, the initial changes that occur following cell activation have not been widely investigated.

It is known that altered epigenetic patterns correlate with the malfunction of effector T cells in tumours [13–16]. Bian *et al*. have demonstrated that methionine metabolism in CD8+ T cells is disrupted in the tumour microenvironment resulting in lowered intracellular methionine levels. Methionine depletion resulted in lowered levels of the methyl donor *S-*adenosylmethionine (SAM) and the loss of histone methylation [17]. During the inflammatory response, neutrophils, and to some extent macrophages, will produce hypochlorous acid (HOCl) by means of the haem-containing enzyme myeloperoxidase (MPO) [18]. HOCl, a cytotoxic oxidant, is a short-lived species in biological systems due to its high reactivity with extracellular methionine, and with amines to form chloramines [19]. Chloramines are cell-permeable, longer-lived oxidants that are likely to be present at sites of infection or inflammation. Chloramines react readily with thiol groups and free methionine to cause cell damage and enzyme inactivation [20–23]. We have previously observed that glycine chloramine (GlyCl) directly inhibits DNA methyltransferase 1 (DNMT1) activity and oxidizes methionine, leading to depletion of the methyl donor SAM [22,24]. This resulted in a global decrease in the maintenance of 5-methylcytosine in proliferating cells without significantly impacting cell viability and was associated with site-specific alterations in DNA methylation and subsequent gene expression. We hypothesized that GlyCl exposure during CD8+ T cell activation may have a similar effect on directing DNA methylation, with relevance to the biological processes that occur in the inflammatory microenvironment of the tumour.

Our investigation assesses the resulting DNA methylation changes of CD8+ T cells between 48 and 96 hours post activation, a pivotal period often overlooked in the existing literature. The effect of GlyCl on directing cell fate by altered DNA methylation was also investigated using this paired sample approach.

## Materials and methods

### Blood collection and separation of CD8+ T cells

Peripheral blood mononuclear cells (PBMCs) were isolated from 50 ml of heparinized blood obtained by venepuncture from eight healthy female volunteers aged 21–25 years with no current infection or history of inflammatory disease. Since immune cell methylation becomes more variable with age we chose to include participants that were close in age. We used women to allow retention of all chromosomes in DNA methylation analyses. Primary CD8+ T cells were isolated from fresh PMBCs by immunomagnetic negative selection using the EasySep™ Human CD8+ T Cell isolation kit (StemCell Technologies), according to the manufacturer’s protocols. This negative selection process involves labelling unwanted cells with antibody complexes and magnetic particles, which are magnetically separated from the untouched cells. The purity of CD8+ T cells was verified by flow cytometry staining with anti-CD8-PE antibody (Beckman Coulter) and was consistently >98%. Red blood cells and monocytes were the major contaminating cell types. Ethical approval for analysis of blood, DNA and DNA methylation from healthy donors was obtained through the University of Otago Ethics Committee (Ref H22/013).

### Glycine chloramine preparation

Glycine chloramine (GlyCl) was prepared as previously described [24]. Briefly, hypochlorous acid was added dropwise to a 10 mM excess of glycine in an equivalent volume of PBS while gently vortexing. The concentration of GlyCl was determined using 5-thio-2-nitrobenzoic acid (TNB) by measuring the change in absorbance at 412 nm, using the molar extinction coefficient for TNB (14; 100 M^–1^ cm^−1^) and adjusting for the 1:2 stoichiometry of the reaction (GlyCl:TNB).

### CD8+ lymphocyte activation and oxidant exposure

**Figure 1.**
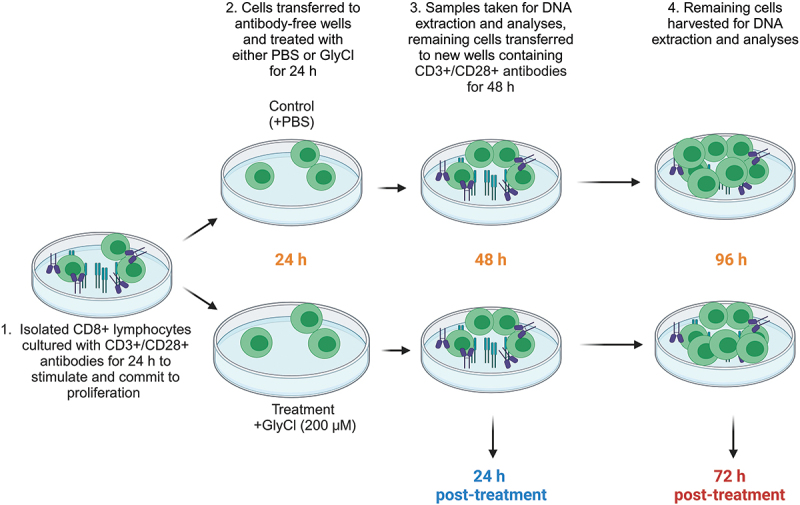
Experimental design.

Isolated CD8+ T cells were cultured in RPMI 1640 + 2 mM L-glutamine + 10% foetal bovine serum (FBS) and 1% penicillin/streptomycin (all from Gibco, Life Technologies), and incubated in 5% CO_2_ at 37°C. Cells were seeded at 10^6^/ml and activated with anti-CD3 (Clone UCHT1), and anti-CD28 (Clone CD28.2) antibodies (Beckman Coulter) for 24 h in 96 well U-bottom tissue culture treated plates (Corning). After initial stimulation, cells were transferred to adjacent wells containing no stimulatory antibodies and media was refreshed so that any change in proliferation or activation could not be attributed to oxidative damage sustained to the CD3/CD28 antibodies as previously described [25]. The cells then received either 200 μM of GlyCl (treatment) or the volumetric equivalent of PBS (control). Twenty-four hours after treatment, samples from each condition were counted and analysed for viability and proliferation. Supernatants and cell pellets were snap frozen and stored at −80º C. The remaining lymphocytes were transferred to adjacent wells containing anti-CD3 and anti-CD28 antibodies and fresh media and were left to proliferate for a further 48 h (72 h post-treatment and 96 h after initial stimulation) (Figure 1). After incubation, the remaining lymphocytes were assessed for viability, growth and proliferation and supernatants and cell pellets were frozen for DNA extraction and subsequent analyses.

CD8+ T cells were isolated from the PBMC layer of the blood of eight healthy volunteers. T cells were stimulated using CD3 and CD28 antibodies for 24 h before being transferred to fresh wells. 200 µM GlyCl was added to the treatment wells, and the control wells received the volumetric equivalent of PBS. Samples were taken 24 h after treatment (48 h after initial stimulation) and 72 h after treatment (96 h after initial stimulation). Created with BioRender.com.

### Cell viability and proliferation

Cell viability was assessed using flow cytometry prior to initial stimulation and then at 24 and 72 h post treatment. The percentage of viable cells was assessed by the exclusion of PI. Growth rate after treatment was determined by live cell counts 24 and 72 h post treatment and expressed as a percent growth (current day count – previous day count)/previous day count x100).

### DNA isolation and bisulfite conversion

DNA was extracted from cell samples using a GeneJet DNA extraction kit (ThermoFisher Scientific, USA) according to the manufacturer’s instructions. Sodium bisulphite treatment of genomic DNA was performed on 1000 ng of DNA using the Zymo Research EZ DNA methylation kit (Zymo Research, Irvine, CA), according to the manufacturer’s recommended protocol for use on the Illumina Infinium MethylationEPIC 850K array (Illumina, Inc., San Diego, CA, USA). Genome-wide DNA methylation profiles were assayed with the Illumina Infinium MethylationEPIC 850k array, at AgResearch Ltd (Invermay, New Zealand). Analysis was performed on all samples in a single batch.

### DNA methylation detection and bioinformatic analysis of DNA methylation

DNA methylation profiles were assessed using the Illumina methylationEPIC v1.0 bead chip array. Methylated and non-methylated values were determined using the minfi pipeline [26], within R. Probes were annotated against the Infinium MethylationEPIC v1.0 B5 Manifest File, which includes genomic coordinates for hg38 and gene regulatory features from Gencode. Data analyses were performed using the Minfi and Limma [27] Bioconductor software packages within the R statistical program (www.R-project.org). All packages, programs and pipelines used in these analyses are freely available, and the workflow was based upon published scripts [28]. Any additional scripts are available from the authors on request. All samples passed quality control together using Subset-quantile Within Array Normalization (SWAN) [29] and variance-stabilizing transformation [30,31]. Probes containing detection p-values >0.05 for 1% or more samples were excluded from further analysis. One sample failed to pass the quality control criteria, and both replicates were removed from the analysis. Because the cell samples were of the same sex (female), all chromosomes were retained; however, probes identified as having polymorphic hybridizing potential and homology to common SNPs [32] were removed. The final data frame contained 808,953 probes available for analysis. Multidimensional scaling of the top 1000 methylation values was performed using pairwise distance method for gene selection, and normalized, filtered methylation values. Hierarchical clustering was performed on a ‘minkowski’ distance matrix calculated using the β-values for all probes, regardless of significance [27].

The methylation status of each probe was calculated using normalized probe signals represented as methylation values (M-values) and β-values. M-values were generated within Minfi as the log_2_ ratio of the signal intensities of methylated probe divided by the unmethylated probe. Unless stated otherwise, statistical analyses were performed using the M-values. β-values (average DNA methylation level for each probe) were used for data visualization and range from 0 (unmethylated) to 1 (methylated). β-values were generated by dividing the methylated probe signal with the sum of the methylated and unmethylated probe signals [33]. Normalized β-values and M-values were manually assessed for fit (Additional file 1: Figure S1).

Differentially methylated positions which are correlated with treatment were identified using a linear regression model within the Limma package, with adjustment for multiple testing. To control for interindividual DNA methylation, paired samples were used from the same participants at both time points and replicate was incorporated as a factor into the statistical design. DNA methylation profiles from the 48 h cell aliquots were subtracted from the 96 h samples. This analysis did not include the cells exposed to GlyCl. The top most significant, differentially methylated CpG positions were identified by significance and were also assessed using a log_2_FC <1.5 in Limma’s topTable algorithm. Adjustment for multiple correction was performed using the ‘Benjamini, Hochberg’ method within Limma and Quantile – Quantile plots for the linear regression model were assessed at each time point using the observed against expected – log10(p-value) (Additional file 2: Figure S1). The observed data show a minor inflation of p-values smaller than expected by chance.

Pathway analysis was performed by comparison gene ontology database (GO) using the Gometh algorithm within the missMethyl R package, with correction for probe bias [34]. Pathway analysis was performed on significant CpG positions that mapped to the regions located from −1500 to −200 base pairs upstream of the transcription start site, and within the first gene exon. The output from the gene ontology analysis was then assessed using the Navigo online algorithm [35], with Resnik semantic similarity-based scoring [36].

### Differentially methylated regions

Differentially methylated regions were interrogated within the Minfi package using the statistical package DMRcate [37]. A methylation differential cut-off of 10 across 5 or more adjacent CpGs was used, and significance was determined using a false discovery rate (FDR) of 0.05.

## Results

### T cell activation and proliferation is associated with DNA methylation change

**Figure 2.**
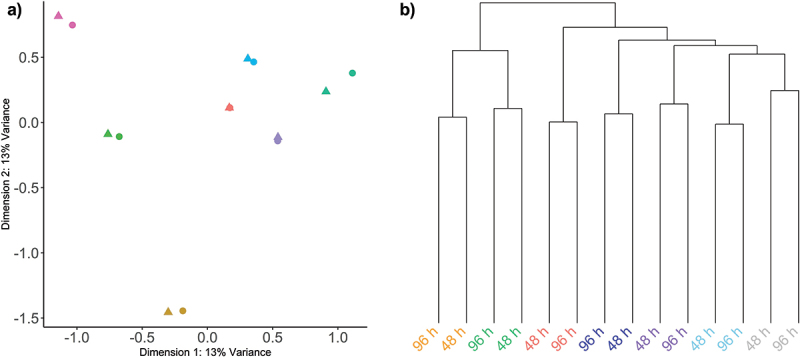
Unsupervised assessment of data variability.

Principle component and hierarchical clustering analysis demonstrated that after 48 h relatively minor DNA methylation changes were observed, and as expected the predominant sources of variation corresponded with interindividual variation (Figure 2). In both instances, DNA methylation profiles were correctly paired with samples from the same participants.

(a) Multidimensional scaling of M-values, with the distances for leading log2FC in dimension 1 represented on the x-axis and the leading log2FC in dimension 2 are represented on the y-axis. Dots represent samples from the 48 h time point and triangles represent samples from the 96 h time point. Samples are coloured by participant. (b) Hierarchical clustering of β-values for all probes. The relative change in β-values is represented on the y-axis and individual samples are represented on the x-axis, samples are coloured according to participant.

**Figure 3.**
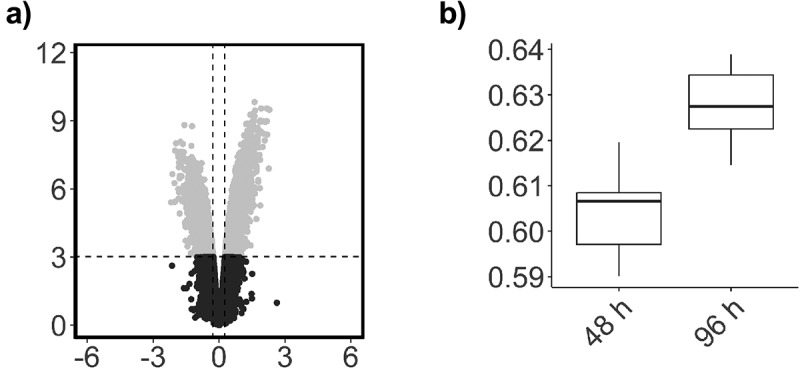
Global DNA methylation (β-values) for all probes.

**Figure 4.**
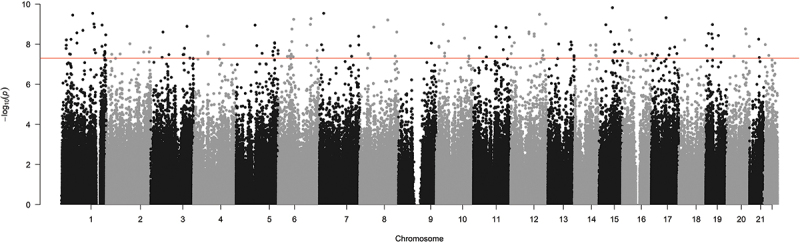
Scatterplot of probe significance ordered by genomic location for all probes across the array, calculated using M-Values.

There was no significant global change in the relative level of DNA methylation across all probes on the array (*p = *0.06, data not shown). Previous analysis indicated that the largest source of variation in DNA methylation was associated with interindividual variation (Figure 2). This was accounted for in our statistical design, which controlled for replication of participants’ samples, and contrasted DNA methylation changes between paired samples at the two time points. There were 15,626 CpGs that demonstrated a significant change between the 48 h and 96 h time points, of which 11,693 were associated with an increase in methylation and 3,933 were associated with a decrease in methylation (Figure 3(a)). The majority of these changes had a relatively small effect size, with only 126 probes demonstrating an effect size greater than a log2 fold change of 1.5. The relative average change in DNA methylation at these 15,626 CpG positions was associated with a significant increase in methylation equivalent to 2.8% between the 48 h and 96 h time points (*p=*0.0005, *t* = 4.7) (Figure 4(b)). To summarize the results, each probe was mapped to the corresponding genomic region and unadjusted significance was plotted across the whole genome as a Manhattan plot. This analysis demonstrated that the significant changes appeared to be concentrated at specific locations across the genome (Figure 4).

(a) Volcano plot with log_2_ fold changes on the *x*-axis versus statistical significance on the *y*-axis -log_10_*P*-value. Grey dots represent probes with an adj. *P*< .05. (b) Relative average methylation levels for each time point. Mean methylation levels for *β-*values are presented as percentages.

All probes are ordered per chromosome position along the x-axis, and p-values as the – log10(p-values) are presented on the y-axis. Genome-wide significance was determined using the ‘Benjamini, Hochberg’ method within Limma and is approximately represented by the horizontal red line.

**Figure 5.**
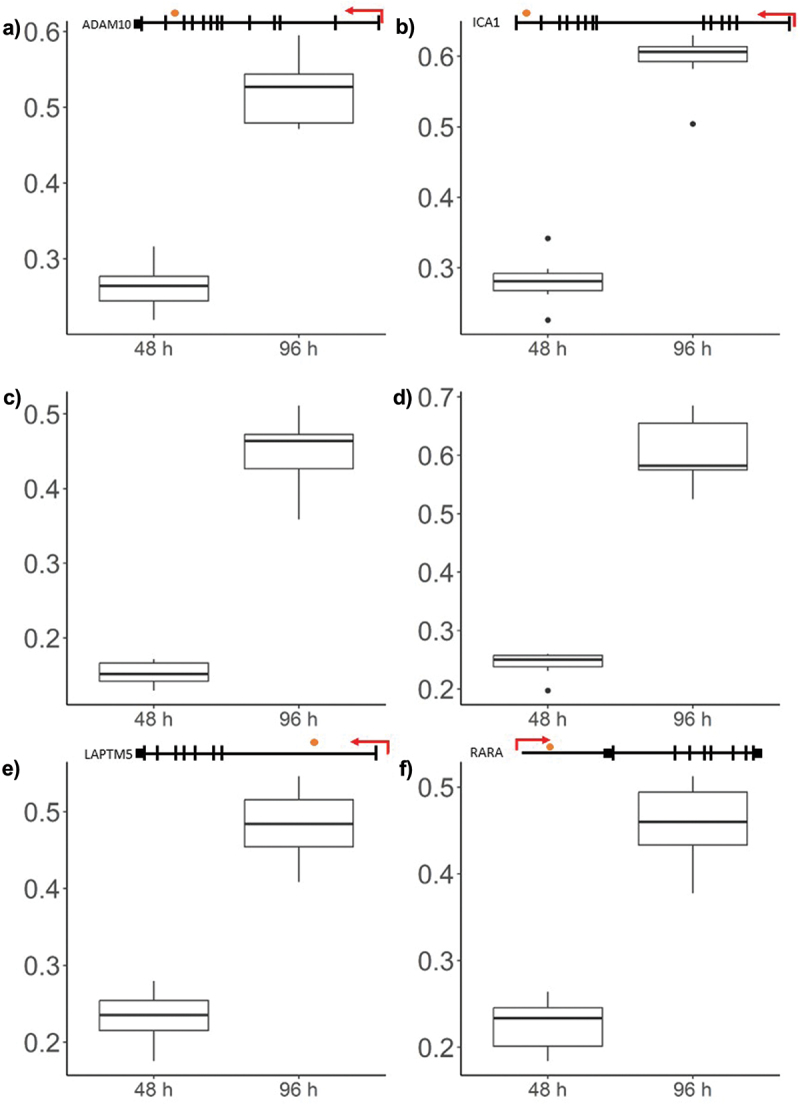
Box plots showing representative β-values at each time point.

The top 20 significantly differentially methylated positions in order of significance are presented in Table 1, which all corresponded with an increase in methylation. To visualize the corresponding change in methylation the raw β-values of the top six most significant CpGs were visualized in Figure 5 and demonstrated an increase in methylation approximately equivalent to 25%.

β-values are represented on the y-axis and time on the x-axis. The CpG sites are marked with an orange dot in the gene scheme placed on top of each graph, with the TSS is marked by a red arrow: a) chr15: 58627950; b) chr7: 8132142; c) chr1: 101264831; d) chr12: 89760982; e) chr1: 30751002; f) chr17: 40310298.

**Table 1.** The top 20 most significant differentially methylated positions, ordered by significance*.

Probe ID	logFC	Adj *p*-val	Chromosome	Location	Gene	Genomic Feature**
cg06555928	1.61	5.31E–05	chr15	58627950	ADAM10	3’UTR
cg27060295	1.91	5.31E–05	chr7	8132142	ICA1	3’UTR
cg09190907	2.17	5.31E–05	chr1	101264831		
cg21870144	2.28	5.31E–05	chr12	89760982		
cg15459165	1.63	5.31E–05	chr1	30751002	LAPTM5	5’UTR
cg13762512	1.55	5.31E–05	chr17	40310298	RARA	5’UTR
cg01725626	1.41	5.31E–05	chr6	110687583	CDK1	Intronic
cg20732076	1.49	5.31E–05	chr6	42367492	TRERF1	5’UTR
cg07781212	1.61	5.31E–05	chr8	86523460	CPNE3	5’UTR
cg22608507	1.60	5.31E–05	chr12	122894760	VPS37B	Intronic
cg24362952	1.87	5.31E–05	chr1	108647018		
cg05679475	1.57	5.31E–05	chr10	17428254	ST8SIA6	5’UTR
cg25060738	2.17	5.31E–05	chr6	107787354	SCML4	5’UTR
cg20641673	2.00	5.31E–05	chr19	16382871	EPS15L1	Intronic
cg27155939	1.52	5.31E–05	chr15	40054961	SRP14-DT	Intronic
cg24219822	2.10	5.31E–05	chr1	160839185	CD244	TSS200
cg20214179	1.85	5.31E–05	chr5	50702804	PARP8	5’UTR
cg17344223	1.44	5.41E–05	chr3	129576937	PLXND1	Intronic
cg14170423	1.30	5.41E–05	chr11	64746336	RASGRP2	TSS1500
cg08964365	1.23	5.41E–05	chr8	38368712	WHSC1L1	5’UTR

*The full dataset of differentially methylated CpGs is available in Supplementary File S1:Table S1.

**UTR = Untranslated Region, TSS = Transcription Start Site (distance in bp).

### Top differentially methylated gene regions

**Figure 6.**
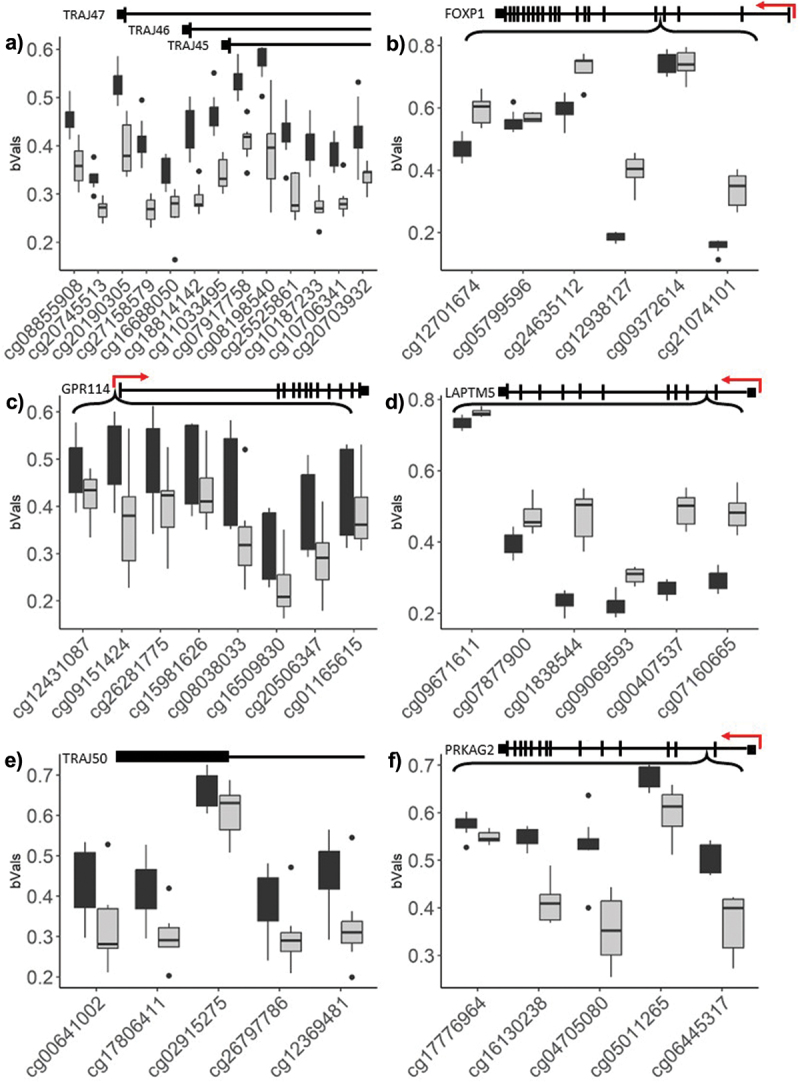
Topmost significant genomic regions displaying differential methylation, across adjacent probe loci.

We next investigated if multiple probes mapping to the same genomic loci demonstrated consistent methylation changes. We limited this analysis to differentially methylated regions (DMRs) that contained five or more adjacent significant CpGs, with an average change in methylation larger than 10%. Under these parameters, we detected seven significant DMRs that differed between the two time points (Table 2), of which six corresponded with annotated gene regions. In contrast with the general trend observed for differentially methylated CpGs, most DMRs demonstrated a decrease in methylation between the 48 and 96 h time points (Figure 6).

Differentially methylated gene regions (DMRs) that displayed a significant change in methylation (y-axis represents β-values) across five or more CpGs (x-axis) between the 48 h (dark grey) and 96 h (light grey) time points. Gene structure is placed on top of each graph, with the TSS is marked by a red arrow. a) *TRAJ44-TRAJ47* b) *FOXP1* c) *GPR114* d) *TBC1D5*, *SATB1* e) *TRAJ50* f) *PRKAG2.*

**Table 2.** Significant DMRs between 48 h and 96 h time points.

Chromosome	Start	End	Width (bp)	CpGs	FDR	Max β-values	Mean β-values	Overlapping genes
chr14	22960834	22964042	3209	13	0.006	−0.192	−0.122	AL132780.3
chr3	71353994	71355303	1310	8	0.009	−0.147	−0.102	FOXP1, AC097634.4
chr16	57576285	57577474	1190	6	0.002	0.230	0.137	ADGRG5
chr3	18459048	18460713	1666	5	0.008	−0.143	−0.109	SATB1-AS1
chr14	22957594	22958402	809	5	0.013	−0.177	−0.112	AL132780.3
chr7	151491913	151493647	1735	6	0.007	−0.348	−0.115	RHEB
chr13	114908155	114909333	1179	6	0.001	0.213	0.112	NA

*Annotated against hg38.

### Gene pathway analysis

The genes corresponding to the topmost significant CpGs were assessed for biological relevance by pathway analysis within the GO databases. This analysis was performed on genes that mapped within gene promoters or the first exon and returned 256 significant molecular pathways. The top pathways for each ontology type are presented in Table 3. When the top most significant CpG sites with a log_2_FC above 1.5 were assessed there were no significant associations with GO terms detected (data not shown). This suggests that relatively small and widespread changes may contribute substantially towards biological outcomes.

**Table 3.** GO pathway terms associated with each ontology type for the genes that demonstrated a significant DNA methylation change, and mapped to promoter regions.

Pathway	ontology	Description	DE	n	adj *p* value
GO:0001775	BP	cell activation	1070	188	1.02E–13
GO:0045321	BP	leukocyte activation	926	168	1.24E–13
GO:0046649	BP	lymphocyte activation	764	146	2.63E–13
GO:0051249	BP	regulation of lymphocyte activation	497	105	5.18E–12
GO:0050865	BP	regulation of cell activation	649	126	5.18E–12
GO:0002694	BP	regulation of leukocyte activation	592	116	2.07E–11
GO:0042110	BP	T cell activation	539	109	2.07E–11
GO:0007165	BP	signal transduction	5887	690	1.17E–10
GO:0046651	BP	lymphocyte proliferation	304	70	1.54E–10
GO:0032943	BP	mononuclear cell proliferation	311	71	1.61E–10
GO:0038023	MF	signaling receptor activity	1448	169	5.35E–05
GO:0060089	MF	molecular transducer activity	1448	169	5.35E–05
GO:0140375	MF	immune receptor activity	140	31	0.000132745
GO:0019955	MF	cytokine binding	139	32	0.00020508
GO:0005126	MF	cytokine receptor binding	270	45	0.000400818
GO:0004888	MF	transmembrane signalling receptor activity	1229	137	0.001570874
GO:0004896	MF	cytokine receptor activity	93	22	0.002898576
GO:0005102	MF	signaling receptor binding	1474	183	0.002964097
GO:0005125	MF	cytokine activity	237	34	0.016452127
GO:0019957	MF	C-C chemokine binding	24	8	0.018652189
GO:0009897	CC	external side of plasma membrane	375	71	4.67E–07
GO:0009986	CC	cell surface	873	135	2.20E–06
GO:0005887	CC	integral component of plasma membrane	1682	222	1.15E–05
GO:0031226	CC	intrinsic component of plasma membrane	1763	231	1.26E–05
GO:0098552	CC	side of membrane	593	96	2.55E–05
GO:0071944	CC	cell periphery	5958	656	4.11E–05
GO:0005886	CC	plasma membrane	5479	606	6.15E–05
GO:0016021	CC	integral component of membrane	5205	544	0.00088294
GO:0031224	CC	intrinsic component of membrane	5370	559	0.001237551
GO:0001772	CC	immunological synapse	45	13	0.048395896

n, number of genes in the pathway.

DE, number of genes that were differentially expressed.

**Figure 7.**
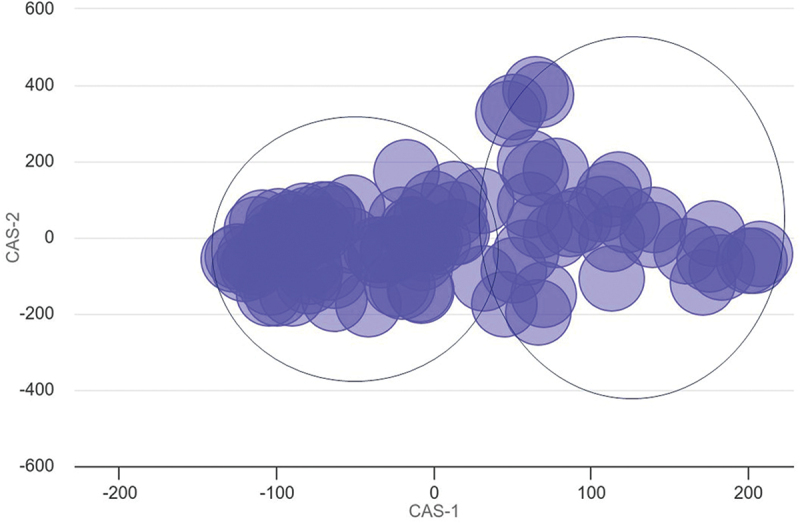
Multidimensional scaling (MDS) plot of top 100 significant pathways.

To summarize the large number of influenced pathways, the top 100 most significant biological processes were investigated for functional similarity and their co-occurrence association [35]. This approach demonstrated that the pathway results approximately separated into two groups (Figure 7). There was a higher density of pathways defined by those that correspond with activation and proliferation, compared against those that correspond with cell signalling and immune response.

Co-occurrence Association Score (CAS) is presented on the x- and y-axis. CAS is a measure used to quantify the frequency of co-occurrences of two GO terms in a single-gene annotation relative to random chance. Pathways contained within the left circle generally correspond with activation and proliferation, whereas pathways within the right circle generally correspond with cell signalling and immune response. The full list of significant pathways is available in (Supplementary file 1: Table S2).

### Effect of glycine chloramine on DNA methylation

Our previous work with T-lymphoma cells demonstrated that exposure to glycine chloramine (GlyCl) corresponded with large genome-wide changes in DNA methylation [24]. We hypothesized that subjecting CD8+ T cells to oxidative stress, akin to that observed during inflammation, could potentially modify DNA methylation patterns during replication. For each participant, the cells were separated into treatment and control samples and exposed to glycine chloramine or PBS. DNA was harvested 24 and 72 h after treatment, and DNA methylation was assessed by comparing the paired treatment and control samples at each time point.

**Figure 8.**
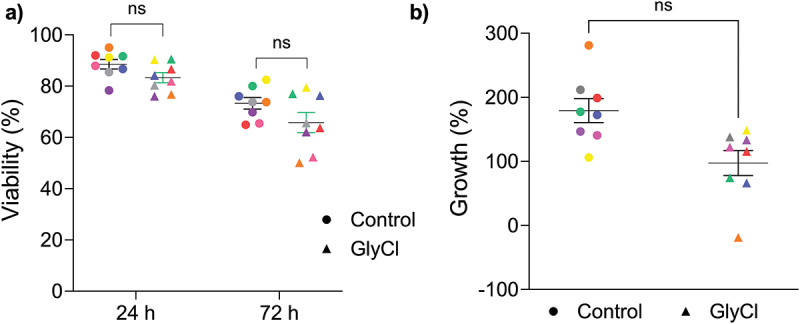
T cell viability and growth.

T cell viability was assessed at 24 and 72 h after treatment by calculating the percentage of cells negative for propidium iodide (PI) by flow cytometry. GlyCl treatment resulted in a small but statistically insignificant decrease in the percentage of live cells at each time point (Figure 8(a)). GlyCl treatment did not appreciably decrease the percentage growth compared to control at either time point (Figure 8(b)).

Cell measures were conducted at 24 and 72 h post-treatment with GlyCl or PBS (Control). Circles represent control samples and triangles represent treatment samples, the colours represent data from the same donor (*n* = 8). Significant differences were determined with paired t-tests. ns, not significant (a) cellular viability after treatment (b) cell growth after treatment.

**Figure 9.**
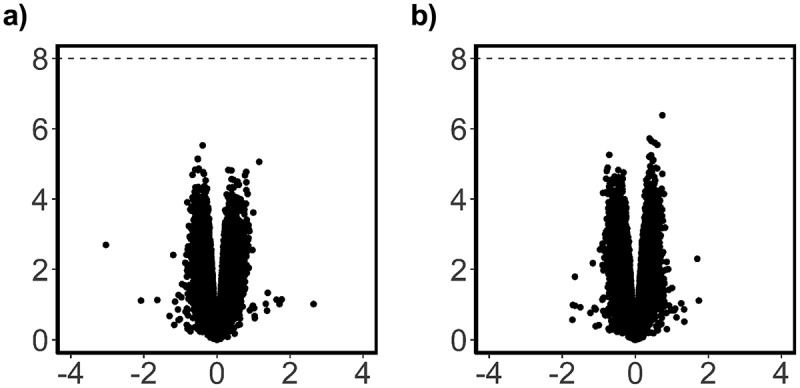
Analysis of overall DNA methylation change due to GlyCl treatment.

We did not detect substantial DNA methylation changes associated with GlyCl changes at either time point, after adjustment for multiple correction (Figure 9). At 24 h, the top most significant probes had an adjusted *p* value of approximately 0.4 and were not investigated further. At the 72 h time point the top probe was associated with the *TRPC6* gene and demonstrated a LogFC of 0.7 (adj *p* = 0.3) (data not shown).

Volcano plots with log2 fold changes on the *x*-axis versus statistical significance on the *y*-axis -log10*P*-value. Black dashed lines approximately represent genome-wide significance probes with an adj. *P* < .05. a) 24 h post-treatment b) 72 h post-treatment.

## Discussion

Our analysis used a paired sample design to investigate the DNA methylation changes that occurred during primary CD8+ T cell activation and proliferation. The CD3/CD28 antibody activation assay used in this study is a common approach used to stimulate lymphocyte proliferation *in vitro*, yet studies that have explored temporal changes in DNA methylation after activation are rare. Based on published literature [17] and our prior research [24,38] we hypothesized that an inflammatory cellular environment may alter the processes of DNA methylation. DNA methylation analysis was performed using the Illumina EPIC850K array, with a robust statistical design that used paired samples at each time point, and accounted for participant replication. Our experimental design used paired samples from seven participants, after one complete experimental replicate was removed after failing quality control. Removing one replicate substantially decreased our statistical power, however, it did not alter the study outcomes.

When comparing T cells 48 and 96 hours after stimulation, there was a general trend of increased methylation following activation, where the top most significant differentially methylated CpGs contribute a significant increase in methylation of ~3%. This effect was spread over 15,626 CpG positions, including only seven DMRs of five or more adjacent CpGs. The resulting DNA methylation levels appeared to be relatively constrained, with no detectable difference in global DNA methylation across the array. In contrast to the trend exhibited by differentially methylated CpGs, most DMRs demonstrated decreased methylation, which is generally associated with the relaxation of the DNA backbone and active transcription. The identification of top differentially methylated genes provides a glimpse into the genomic landscape most responsive to T cell activation. The top methylation changes corresponded to significant increased methylation and occurred at genes including *ADAM10*, *ICA1*, and *LAPTM5*, which have known roles in immune regulation of T-cells. Increased methylation at these sites may have led to decreased expression of these genes that would likely result in an increase in the cytotoxic capacity of the CD8+ T cell. For example, *ADAM10* codes for ‘a disintegrin and metalloprotease’ (ADAM) protease that modulates the cell membrane expression of the Fas ligand, a major effector molecule of cytotoxic T lymphocytes [39]. ADAM10 cleavage of the Fas ligand has been shown to counter the killing activity of T cells [40]. LAPTM5 is involved in the lysosomal degradation of the CD3 receptor which negatively affects activation in both T cells and B cells [41,42]. *ICA1* codes for auto-antigen and is most highly expressed in T regulatory cells, suggesting that activation in our model shifted DNA methylation profiles towards a phenotype with effector T cell attributes [43].

The top most significant biological processes all related to T cell activation and proliferation, interestingly the changes of largest effect size were not strongly associated with known biological processes. The seemingly modest effect sizes of methylation changes underscore the importance of nuanced epigenetic modifications in directing immune responses and the precision with which T cell activation orchestrates epigenetic modifications. The non-random distribution of these changes across the genome warrants further investigation. These findings bear significant implications for our understanding of immune regulation, hinting at a targeted and context-dependent nature of DNA methylation alterations during T cell activation. These results posit that even small and widespread changes in DNA methylation might wield considerable influence over biological outcomes and in shaping the immune landscape.

CD8+ lymphocytes were treated with a sublethal dose of glycine chloramine 24 h after initial stimulation to target the time point when the cells would be rapidly replicating and remethylating their DNA prior to first cell division. It was surprising that GlyCl treatment did not result in a significant reduction in proliferation in these cells, which has previously been observed in a similar experimental system using activated neutrophils [44]. We also did not observe any DNA methylation changes unique to GlyCl exposure, which suggests that once the CD8+ T-cells have committed to proliferation, early events may occur in the cell that protect against mild oxidative stress and methionine depletion. T cells possess robust mechanisms to guard against disruptions by environmental stressors. Upon stimulation, T cells undergo rapid upregulation of the methionine transporter Slc7a5, which imports methionine for use in protein synthesis and can be converted into SAM [45]. It is possible that the T cells in our model were able to increase methionine uptake more rapidly than expected, such that depletion of extracellular methionine through exposure to GlyCl was ineffective at sufficiently reducing methionine levels within the cell [45]. Our previous work in T-lymphoma cells demonstrated that GlyCl can also impair DNA methylation by directly inhibiting DNMT1 activity [22,24] and other studies have shown that DNMT activity is a requirement for murine CD8+ T cell proliferation and effector phenotype differentiation [46]. We therefore speculate that the concentration of GlyCl in our system was insufficient to both deplete methionine and inhibit DNMT activity in CD8+ T cells at the time points analysed. More extensive investigations of the impact of GlyCl on CD8+ T cells is warranted. Despite T cells being stimulated at the same time, our previous work was performed on cell cycle synchronized T-lymphoma cells which allows for more confidence about the timing of DNA replication. Our hypothesis was partially based on previous work that demonstrated methionine depletion through co-culture with tumour cells had a significant effect on histone methylation in CD8+ T cells [17], however histone methylation was not assessed in our study. It is feasible that chromatin conformational changes at our chosen time points rendered the DNA inaccessible during GlyCl exposure. Investigating histone methylation changes in the context of chloramine exposure or in a neutrophil co-culture model may be a more important aspect of the epigenetic response in CD8+ T cells under inflammatory conditions.

Epigenetic processes are central to T cell lineage decisions but most studies have focussed on the impact of histone modifications in disease and infection models [47,48] and DNA methylation studies of CD8+ T cell activation using CD3/CD28 stimulation models have been conducted primarily in mice [46,49]. Our study has a unique emphasis on the dynamics of DNA methylation during the critical early hours of T cell activation. This novel temporal perspective adds a layer of complexity to the understanding of epigenetic regulation in the context of immune responses.

## Conclusion

Our investigation into CD8+ T cell activation and DNA methylation dynamics presents new insights into the epigenetic events steering stimulatory responses. The activation of CD8+ T cells through CD28 and CD3 antibodies instigates a cascade of significant alterations in DNA methylation profiles. The observed changes, though subtle, may orchestrate important downstream effects in the immune response. Understanding the epigenetic regulation of T cells soon after activation contributes valuable insights to the broader field of immunology and opens avenues for future investigations into the development of targeted therapies that can recalibrate immune responses in the context of various diseases.

## Supplementary Material

Supplementary_File1_S1.xlsx

Supplementary_File_S2.xlsx

Additional_File_2_Fig.1_qq_Activation.jpg

Additional_file_1_Fig1_.pdf

## Data Availability

The authors confirm that the analysed data supporting the findings of this study are available within the article [and/or] its supplementary materials. Request for raw data files can be made available from the corresponding author AJS upon reasonable request; however, these represent private data and are not available for public use.
