# Supplementary figures and images for: Widespread genomic de novo DNA methylation occurs following CD8+ T cell activation and proliferation
Source: Epigenetics. 2024 Jun 20;19(1):2367385. doi: 10.1080/15592294.2024.2367385 (PMC11195465; doi:10.1080/15592294.2024.2367385)

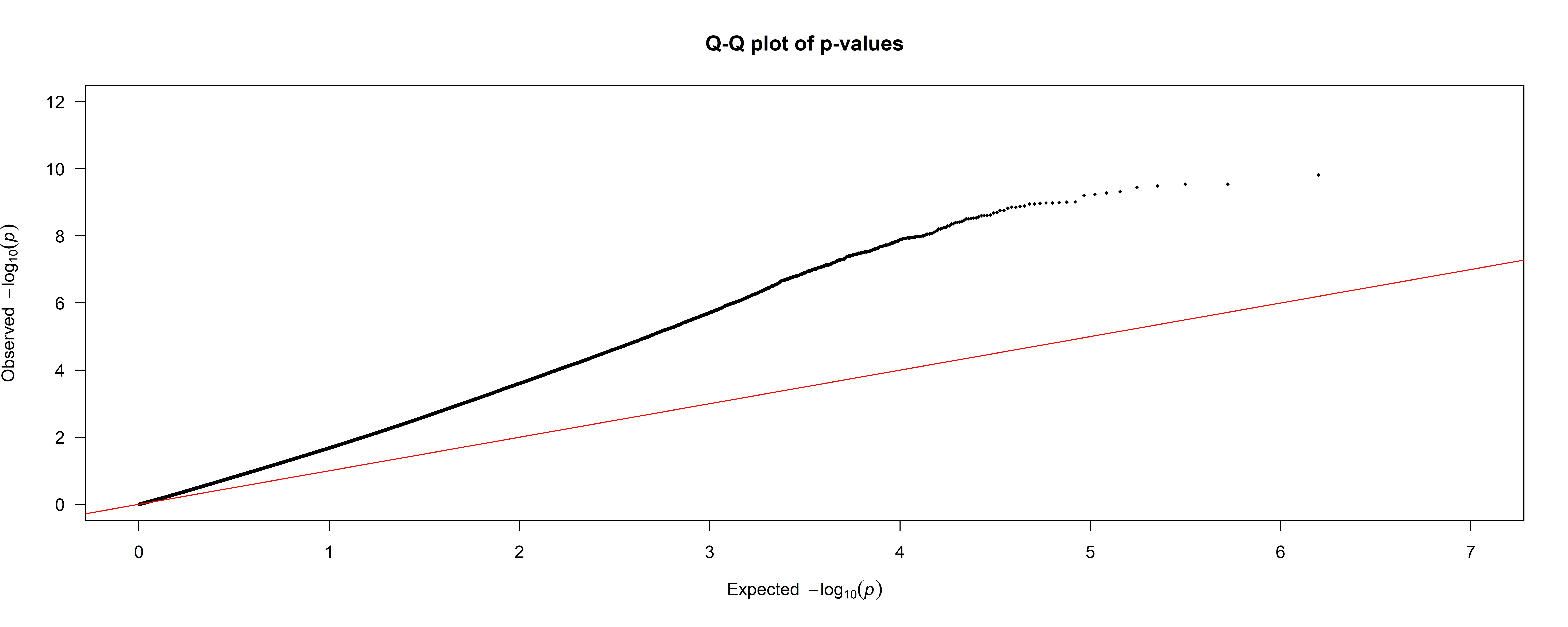

Supplement: Additional_File_2_Fig.1_qq_Activation.jpg [file KEPI_A_2367385_SM9848.jpg]
