# Supplementary material for: Widespread genomic de novo DNA methylation occurs following CD8+ T cell activation and proliferation
Source: Epigenetics. 2024 Jun 20;19(1):2367385. doi: 10.1080/15592294.2024.2367385 (PMC11195465; doi:10.1080/15592294.2024.2367385)
Supplement: Additional_file_1_Fig1_.pdf [file KEPI_A_2367385_SM9847.pdf]

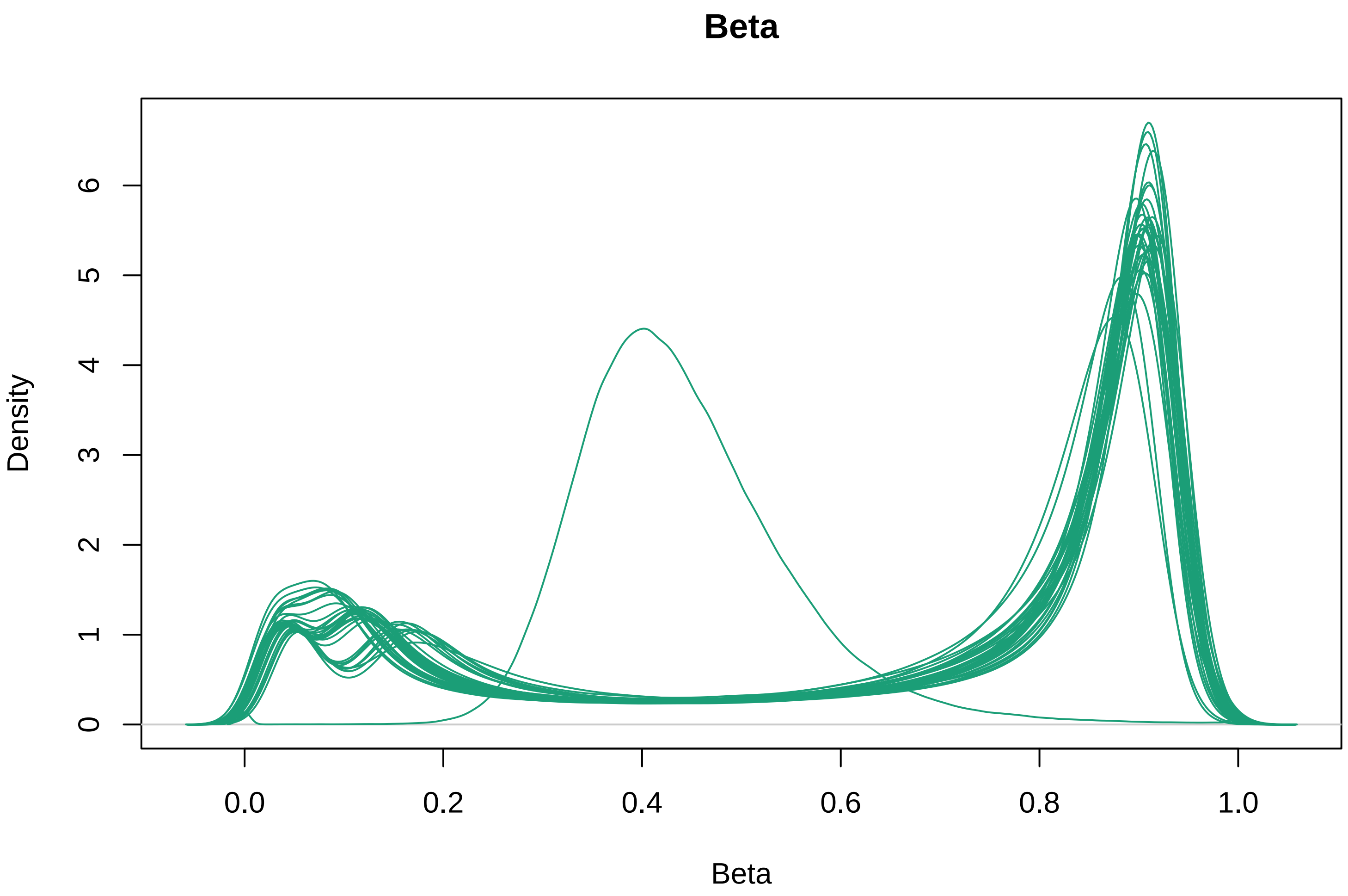
Beta
Density
0
1
2
3
4
5
6
0.0
0.2
0.4
0.6
0.8
1.0
Beta

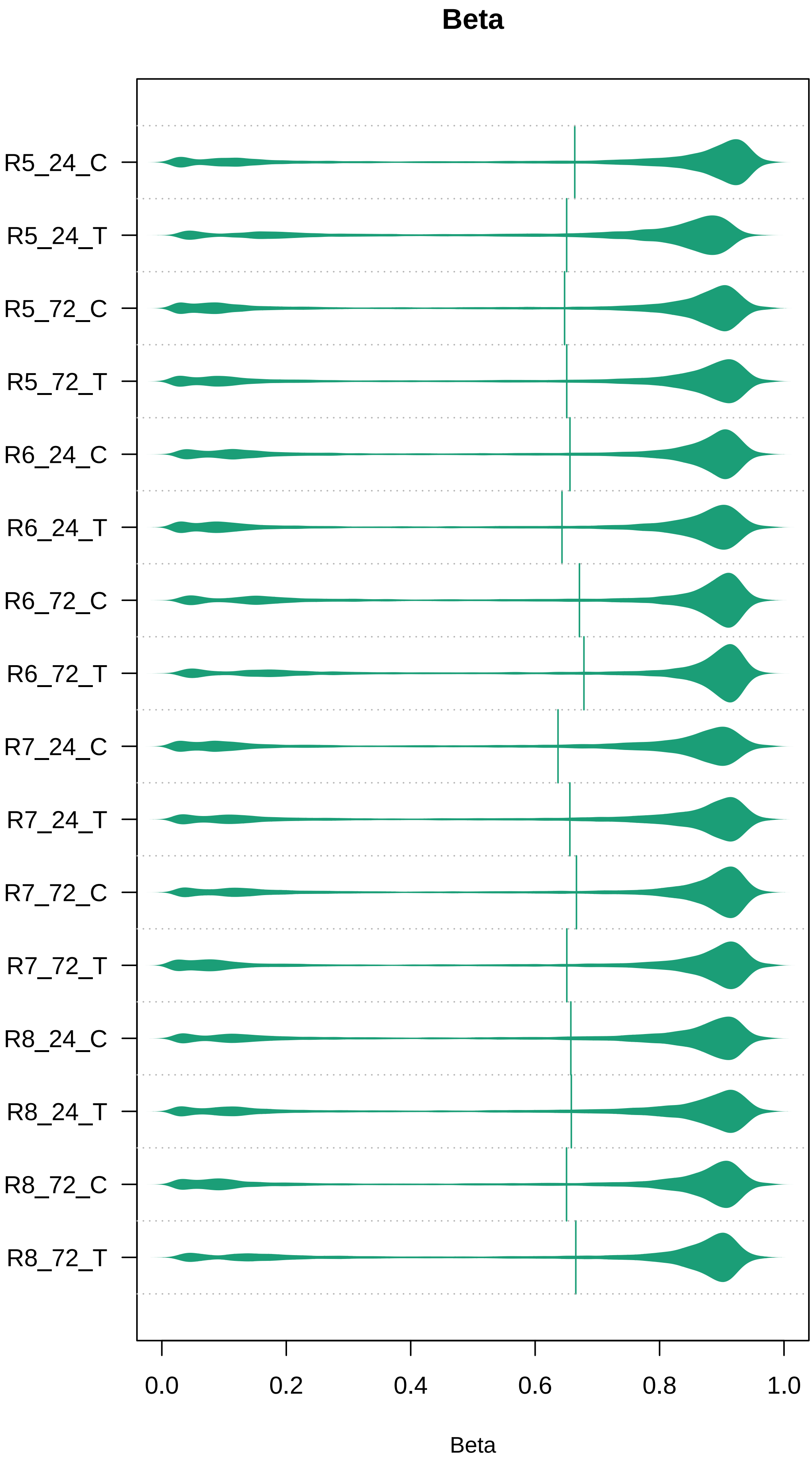
Beta
R5_24_C
R5_24_T
R5_72_C
R5_72_T
R6_24_C
R6_24_T
R6_72_C
R6_72_T
R7_24_C
R7_24_T
R7_72_C
R7_72_T
R8_24_C
R8_24_T
R8_72_C
R8_72_T
0.0
0.2
0.4
0.6
0.8
1.0
Beta

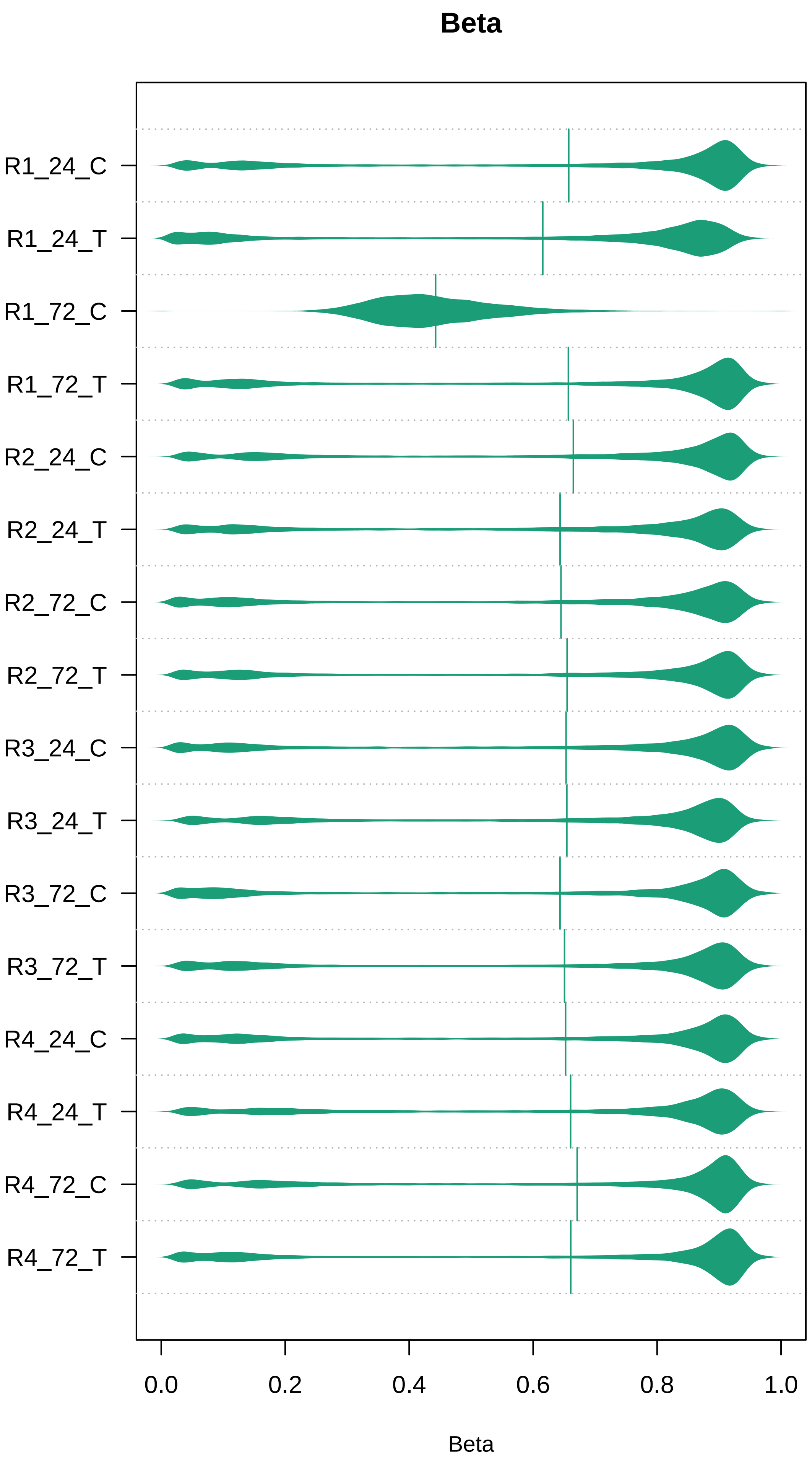

Beta
R1_24_C
R1_24_T
R1_72_C
R1_72_T
R2_24_C
R2_24_T
R2_72_C
R2_72_T
R3_24_C
R3_24_T
R3_72_C
R3_72_T
R4_24_C
R4_24_T
R4_72_C
R4_72_T
0.0
0.2
0.4
0.6
0.8
1.0
Beta

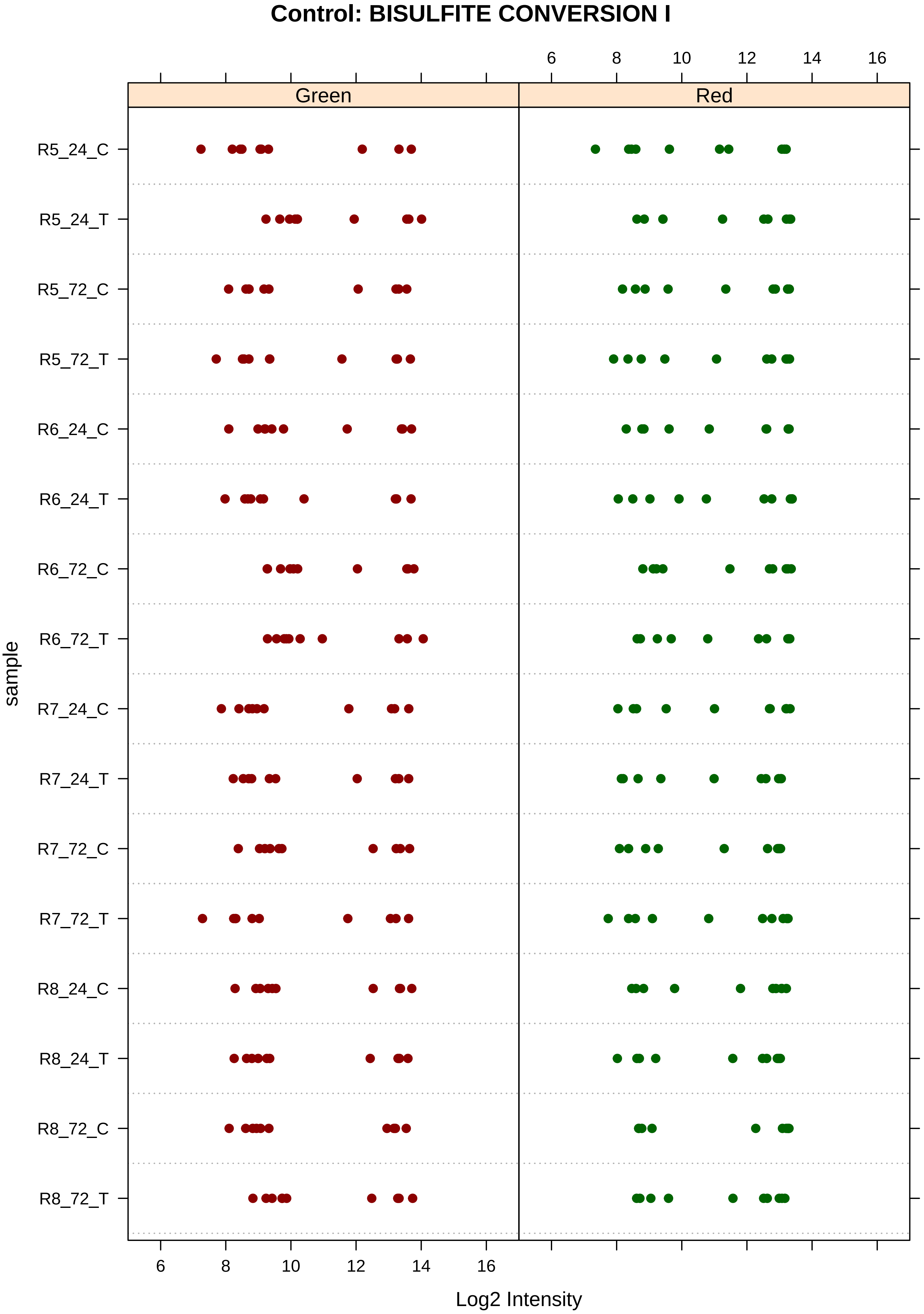
Control: BISULFITE CONVERSION I
Green
Red
R5_24_C
R5_24_T
R5_72_C
R5_72_T
R6_24_C
R6_24_T
R6_72_C
R6_72_T
R7_24_C
R7_24_T
R7_72_C
R7_72_T
R8_24_C
R8_24_T
R8_72_C
R8_72_T
sample
6
8
10
12
14
16
Log2 Intensity

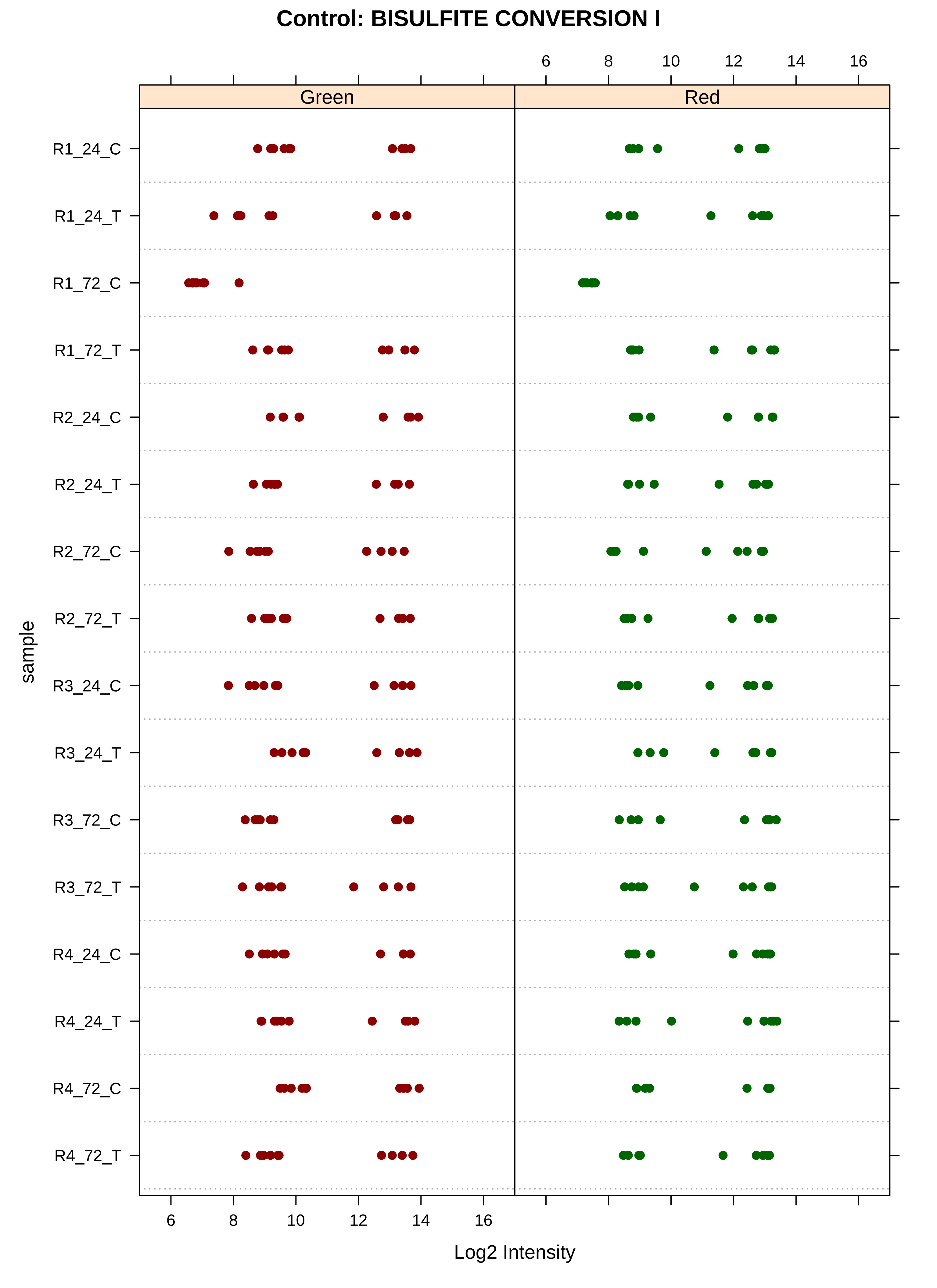
Control: BISULFITE CONVERSION I
Green
Red
sample
Log2 Intensity
R1_24_C
R1_24_T
R1_72_C
R1_72_T
R2_24_C
R2_24_T
R2_72_C
R2_72_T
R3_24_C
R3_24_T
R3_72_C
R3_72_T
R4_24_C
R4_24_T
R4_72_C
R4_72_T
6
8
10
12
14
16

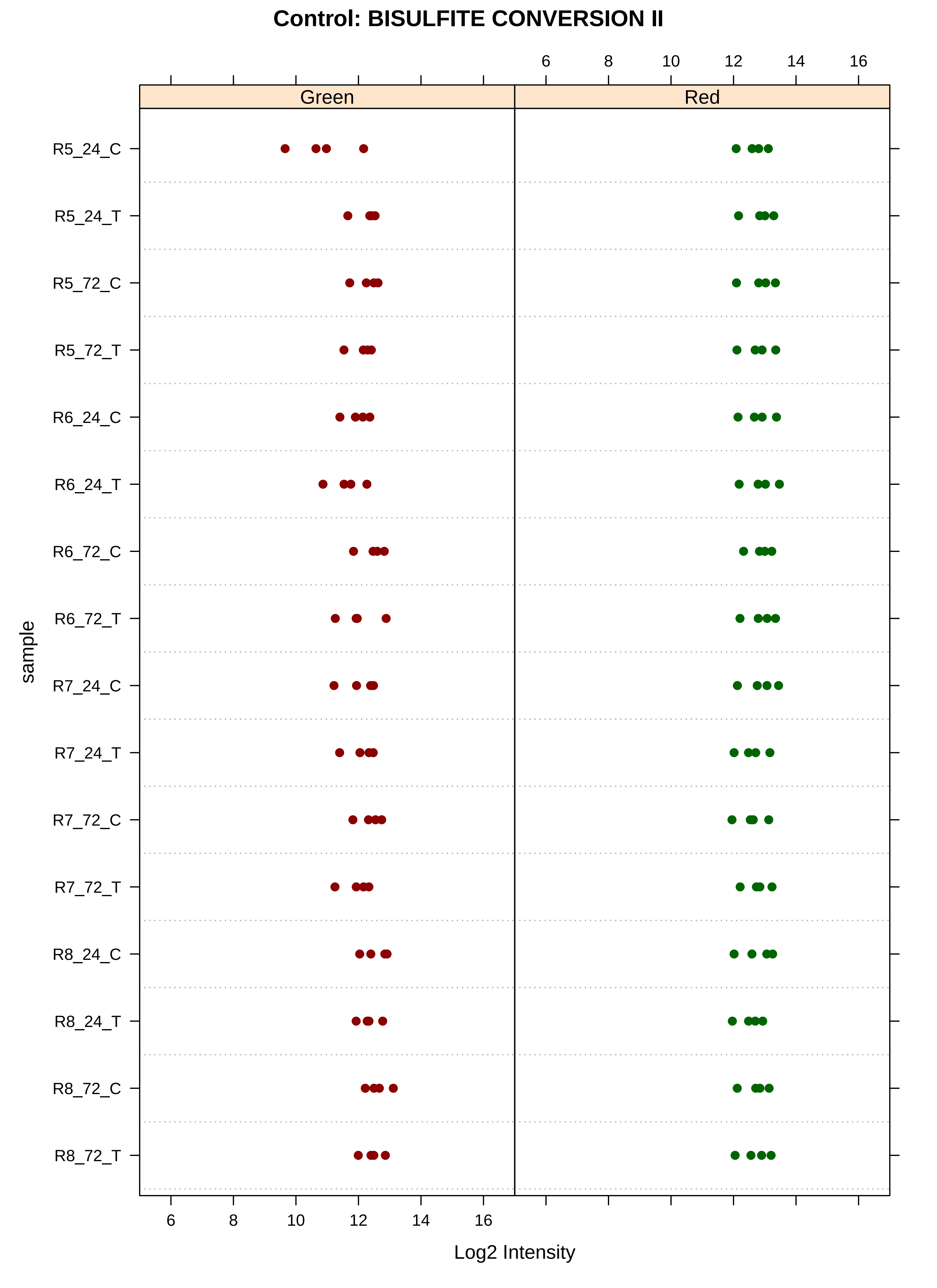

Control: BISULFITE CONVERSION II
Green
Red
sample
R5_24_C
R5_24_T
R5_72_C
R5_72_T
R6_24_C
R6_24_T
R6_72_C
R6_72_T
R7_24_C
R7_24_T
R7_72_C
R7_72_T
R8_24_C
R8_24_T
R8_72_C
R8_72_T
6
8
10
12
14
16
Log2 Intensity

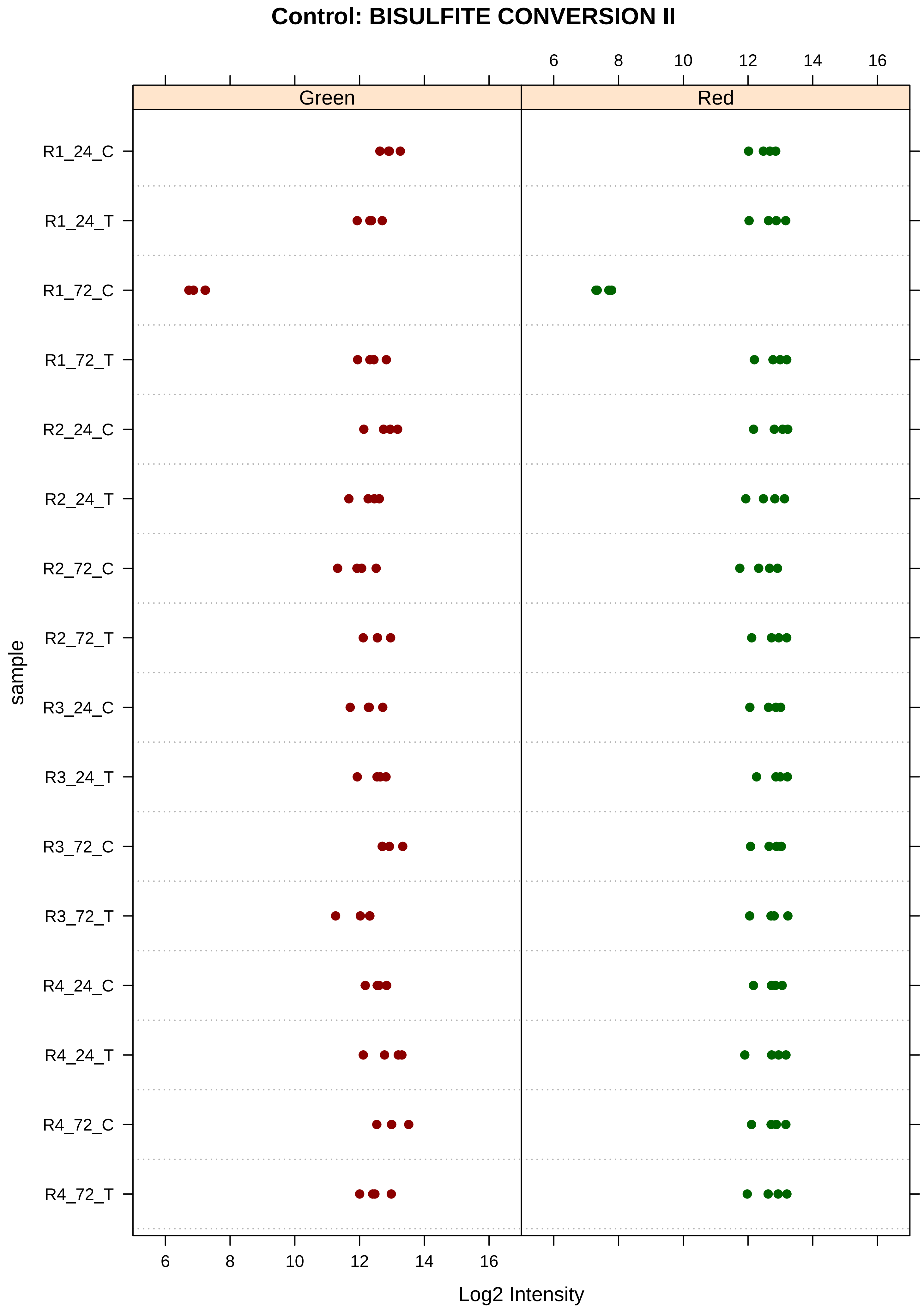

Control: BISULFITE CONVERSION II
Green
Red
sample
R1_24_C
R1_24_T
R1_72_C
R1_72_T
R2_24_C
R2_24_T
R2_72_C
R2_72_T
R3_24_C
R3_24_T
R3_72_C
R3_72_T
R4_24_C
R4_24_T
R4_72_C
R4_72_T
6
8
10
12
14
16
Log2 Intensity

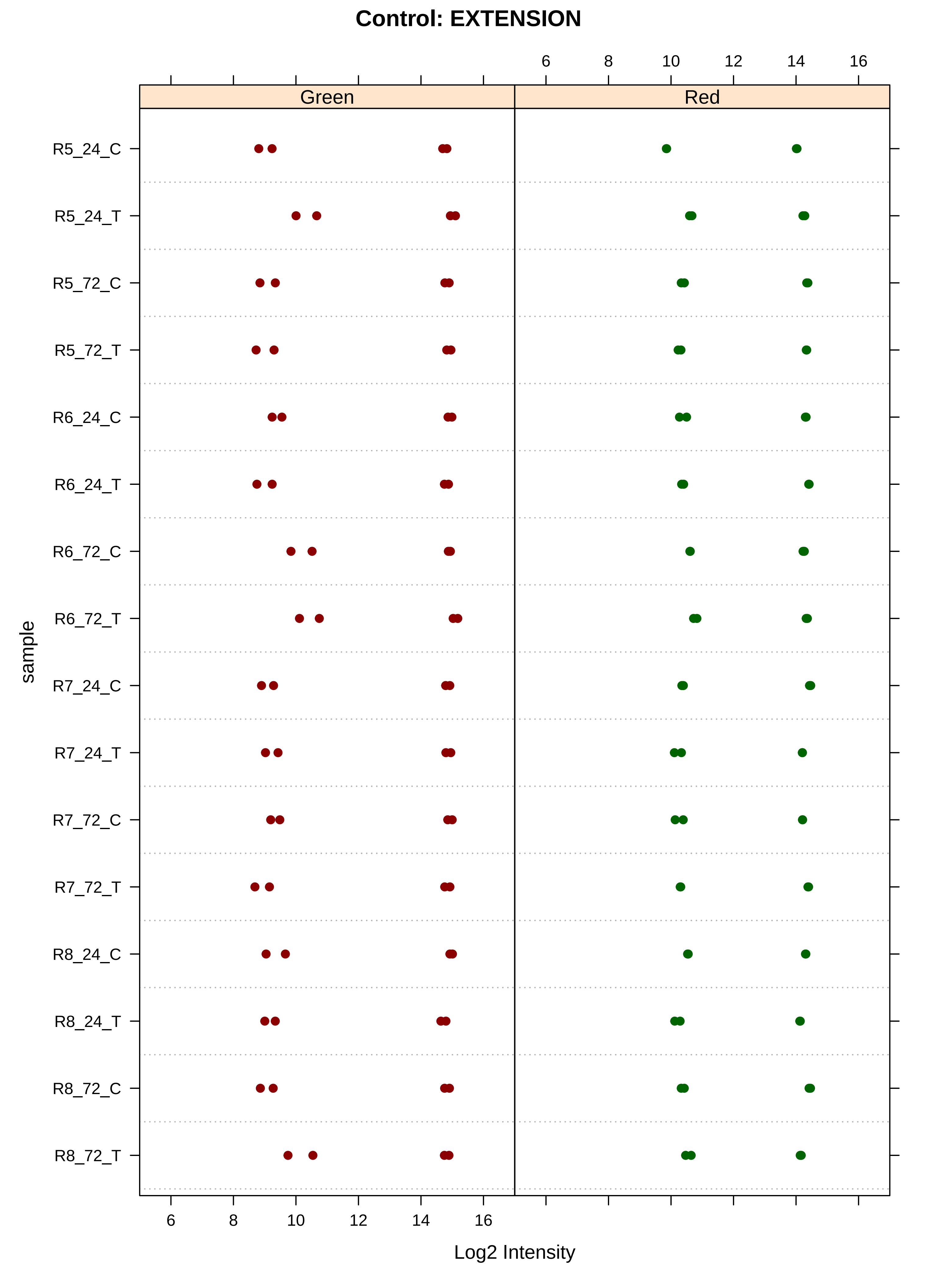

Control: EXTENSION
Green
Red
sample
R5_24_C
R5_24_T
R5_72_C
R5_72_T
R6_24_C
R6_24_T
R6_72_C
R6_72_T
R7_24_C
R7_24_T
R7_72_C
R7_72_T
R8_24_C
R8_24_T
R8_72_C
R8_72_T
6
8
10
12
14
16
Log2 Intensity

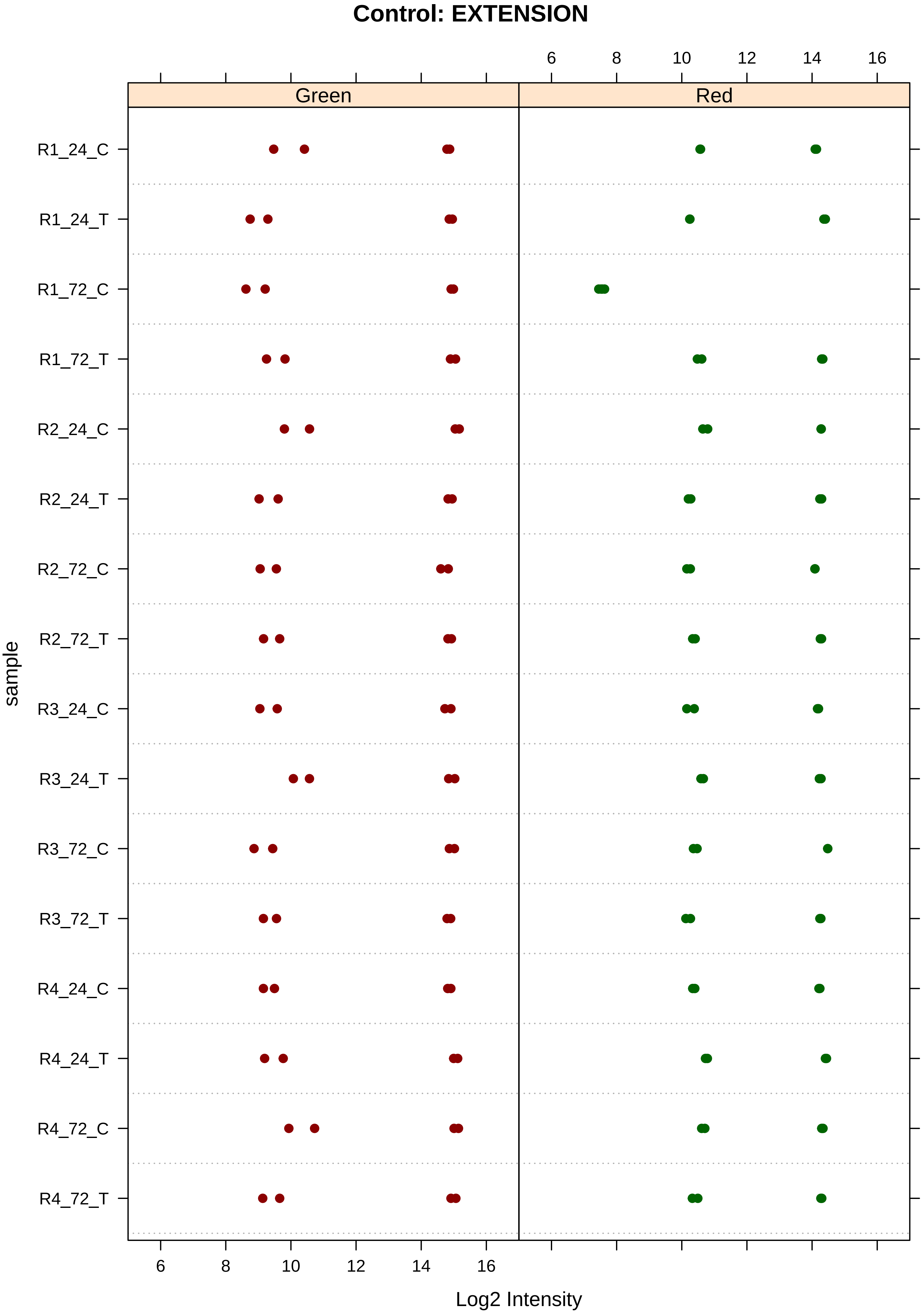

Control: EXTENSION
Green
Red
sample
R1_24_C
R1_24_T
R1_72_C
R1_72_T
R2_24_C
R2_24_T
R2_72_C
R2_72_T
R3_24_C
R3_24_T
R3_72_C
R3_72_T
R4_24_C
R4_24_T
R4_72_C
R4_72_T
6
8
10
12
14
16
Log2 Intensity

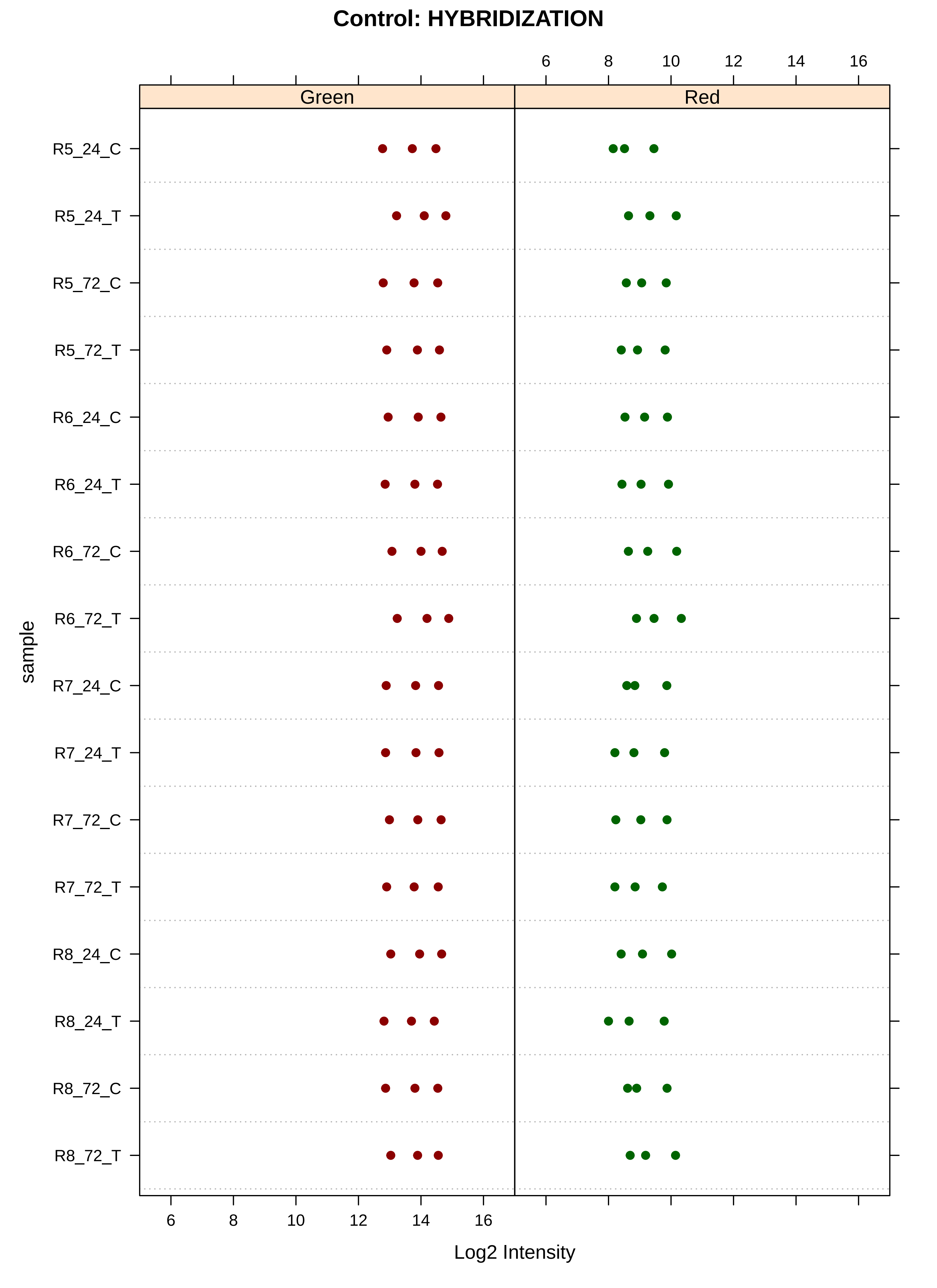

Control: HYBRIDIZATION
Green
Red
sample
Log2 Intensity
R5_24_C
R5_24_T
R5_72_C
R5_72_T
R6_24_C
R6_24_T
R6_72_C
R6_72_T
R7_24_C
R7_24_T
R7_72_C
R7_72_T
R8_24_C
R8_24_T
R8_72_C
R8_72_T
6
8
10
12
14
16

Control: HYBRIDIZATION

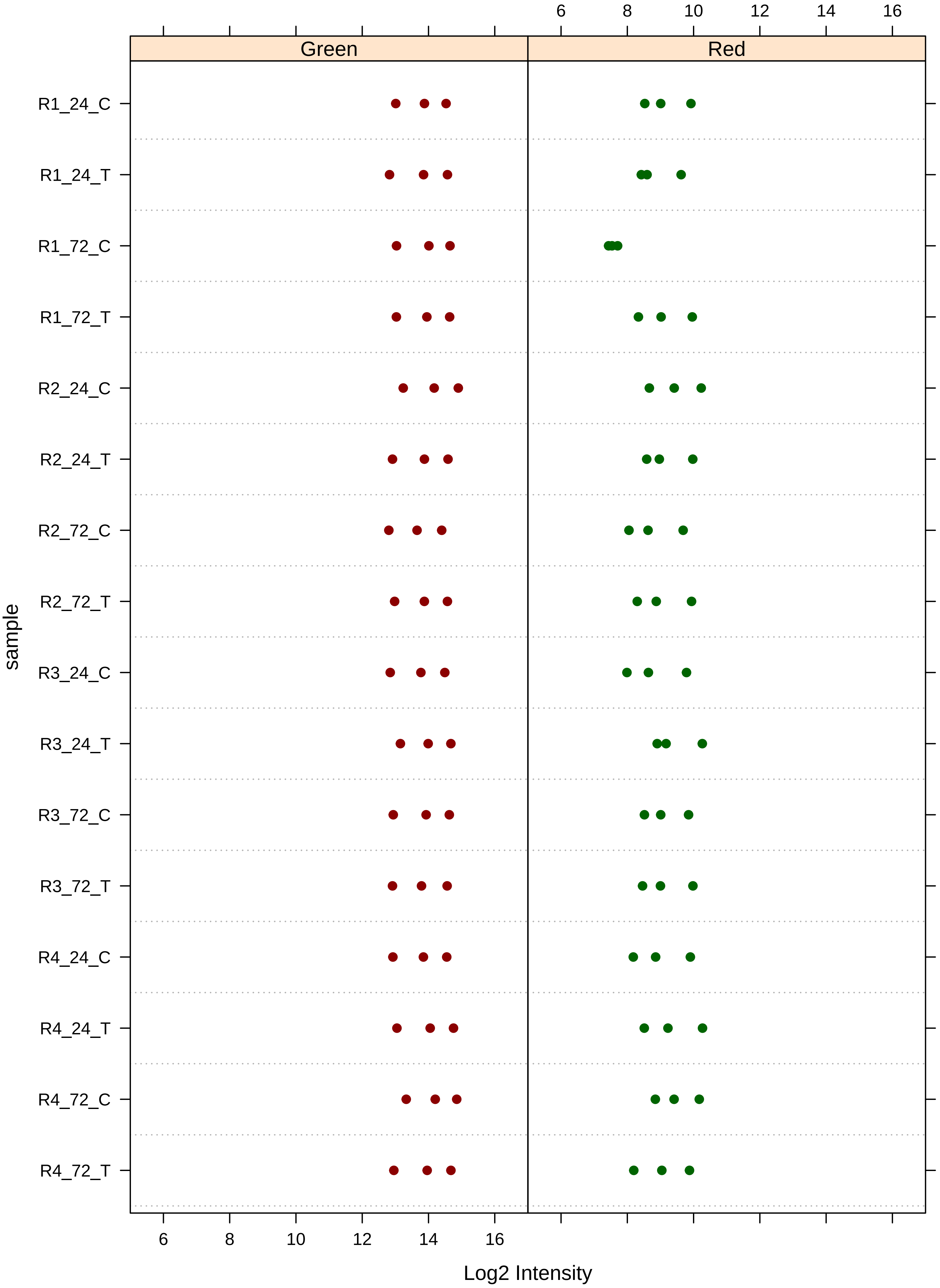

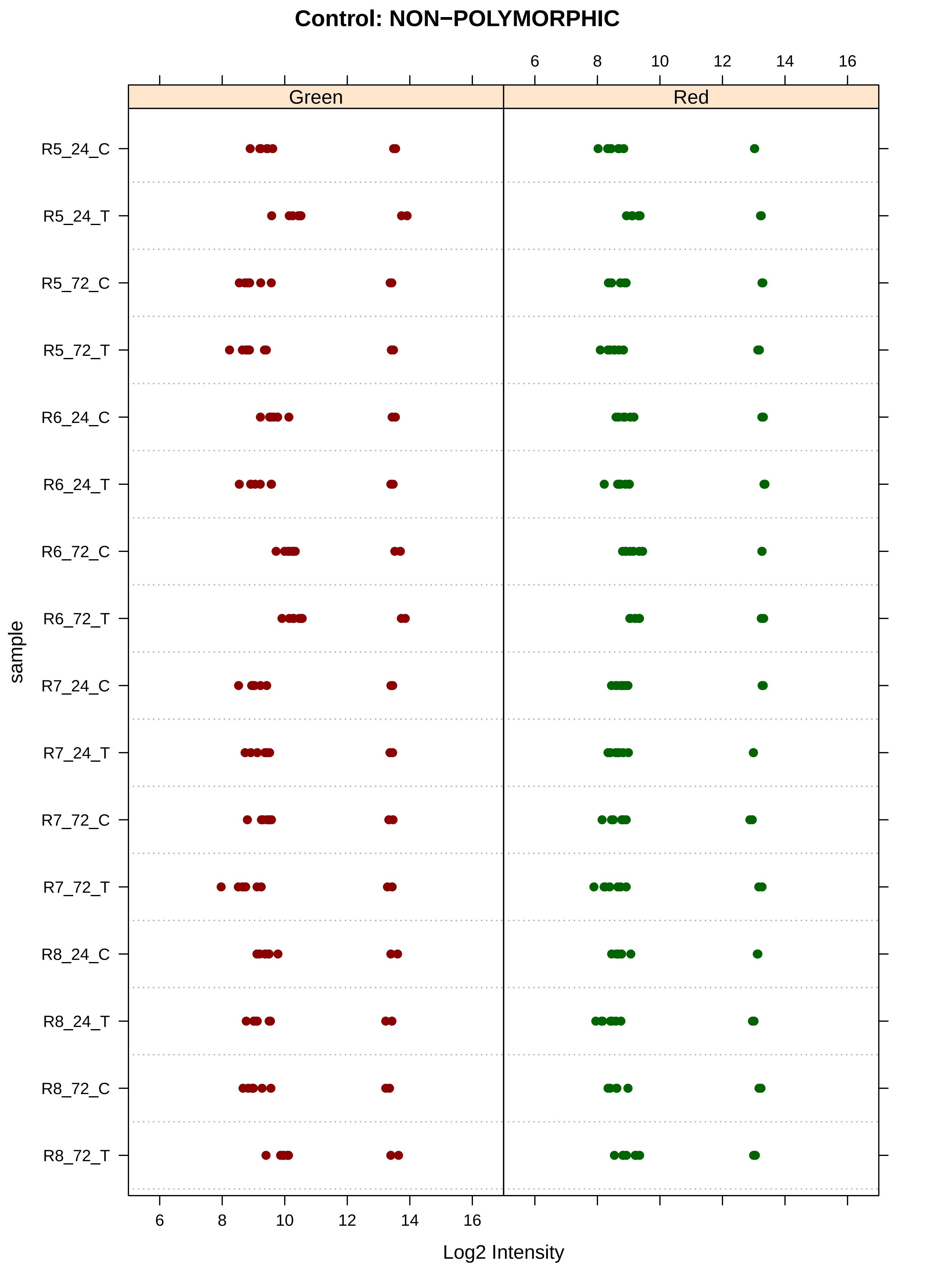

Control: NON−POLYMORPHIC
Green
Red
sample
Log2 Intensity
R5_24_C
R5_24_T
R5_72_C
R5_72_T
R6_24_C
R6_24_T
R6_72_C
R6_72_T
R7_24_C
R7_24_T
R7_72_C
R7_72_T
R8_24_C
R8_24_T
R8_72_C
R8_72_T
6
8
10
12
14
16

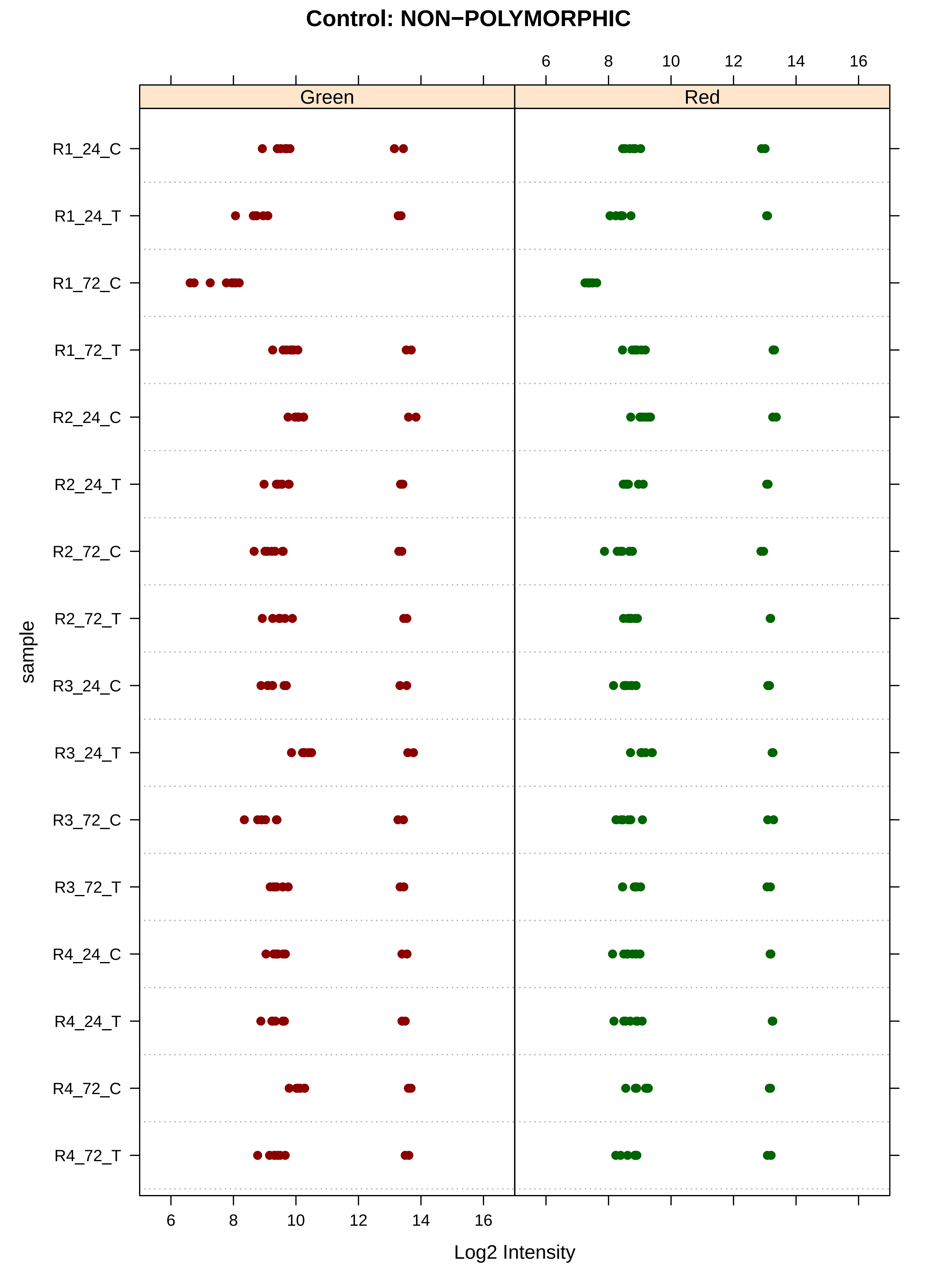
Control: NON−POLYMORPHIC
Green
Red
sample
Log2 Intensity
R1_24_C
R1_24_T
R1_72_C
R1_72_T
R2_24_C
R2_24_T
R2_72_C
R2_72_T
R3_24_C
R3_24_T
R3_72_C
R3_72_T
R4_24_C
R4_24_T
R4_72_C
R4_72_T
6
8
10
12
14
16

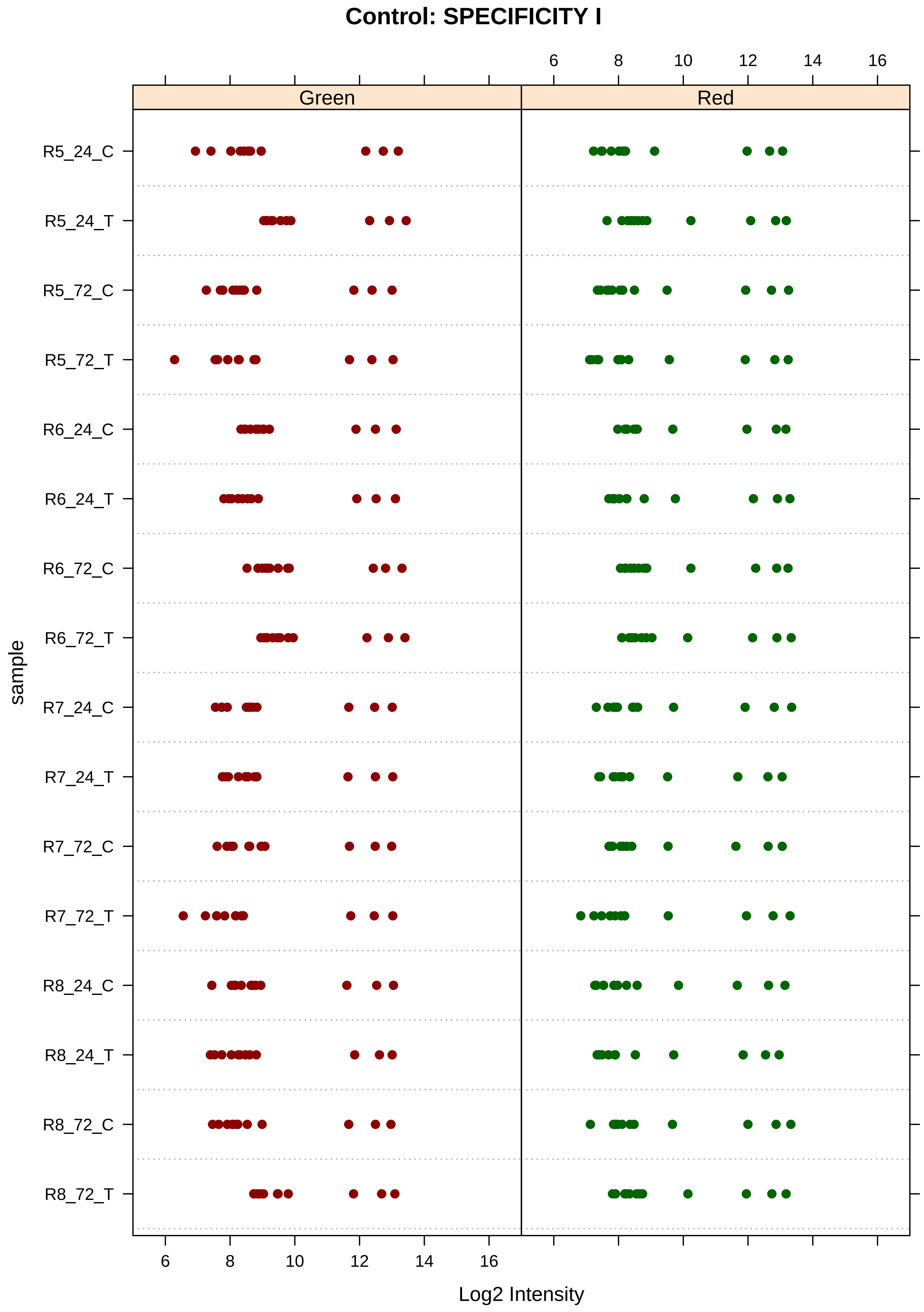

Control: SPECIFICITY I
Green
Red
sample
Log2 Intensity
R5_24_C
R5_24_T
R5_72_C
R5_72_T
R6_24_C
R6_24_T
R6_72_C
R6_72_T
R7_24_C
R7_24_T
R7_72_C
R7_72_T
R8_24_C
R8_24_T
R8_72_C
R8_72_T
6
8
10
12
14
16

Control: SPECIFICITY I

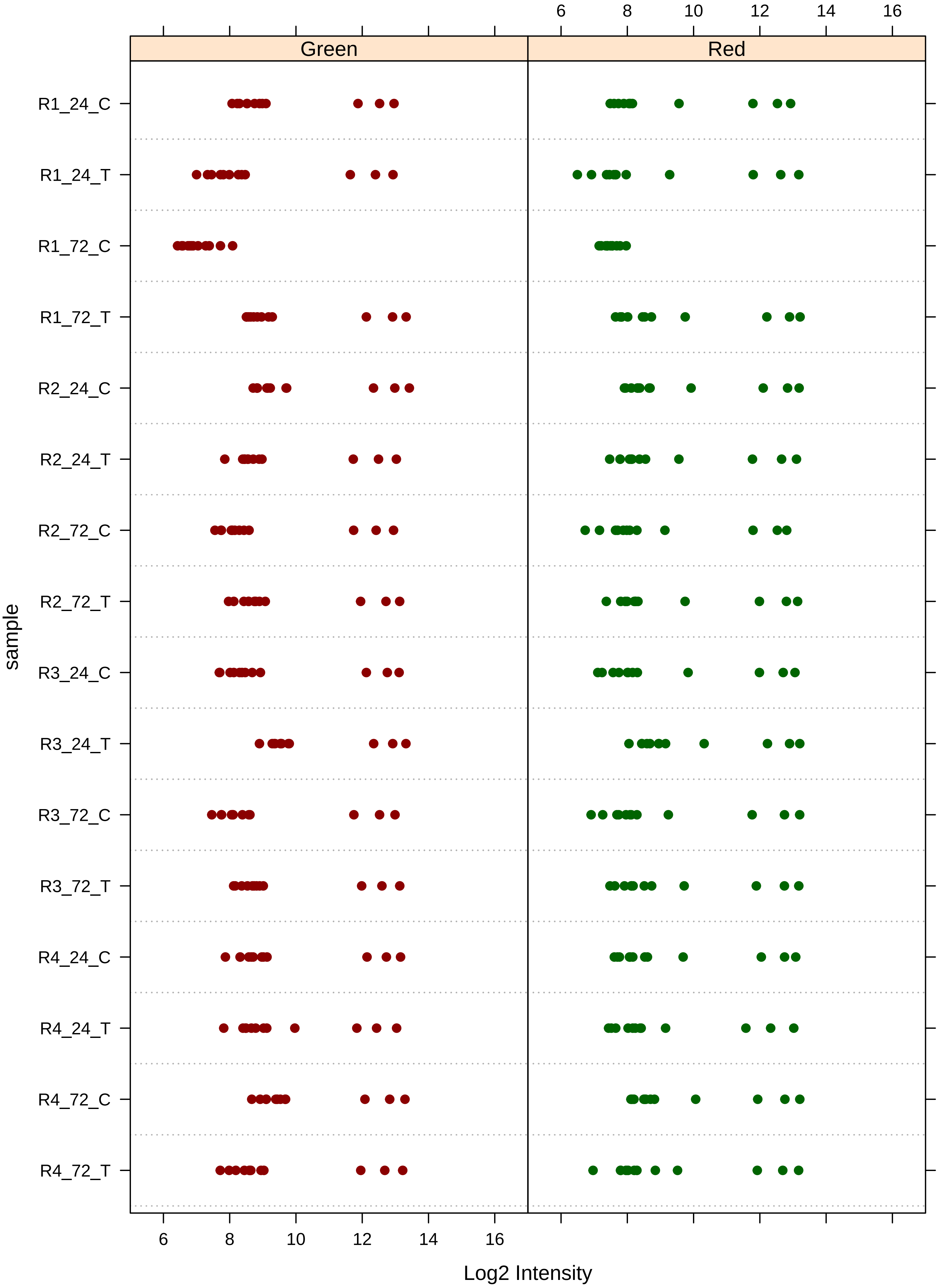

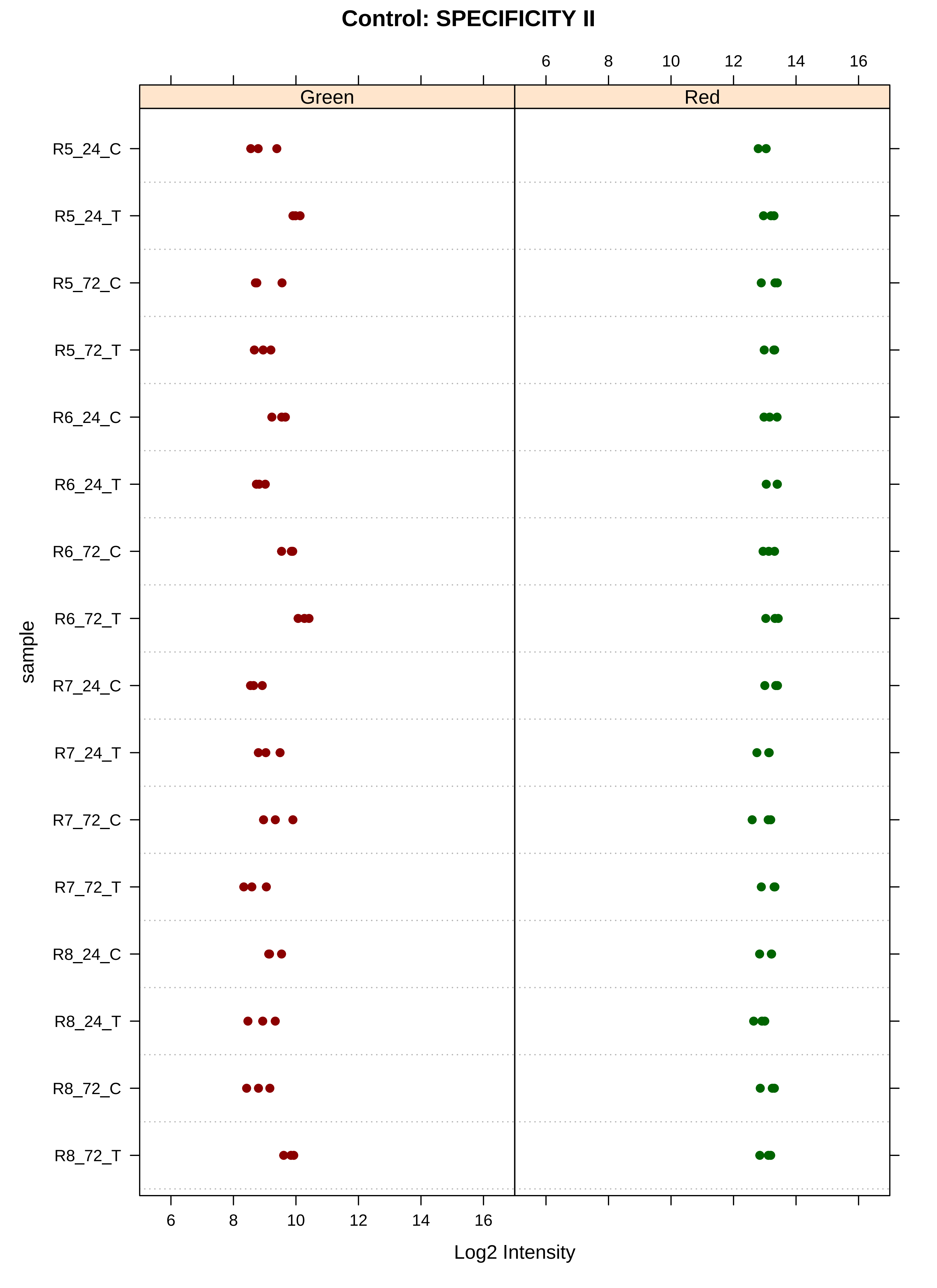
Control: SPECIFICITY II
Green
Red
sample
R5_24_C
R5_24_T
R5_72_C
R5_72_T
R6_24_C
R6_24_T
R6_72_C
R6_72_T
R7_24_C
R7_24_T
R7_72_C
R7_72_T
R8_24_C
R8_24_T
R8_72_C
R8_72_T
6
8
10
12
14
16
Log2 Intensity

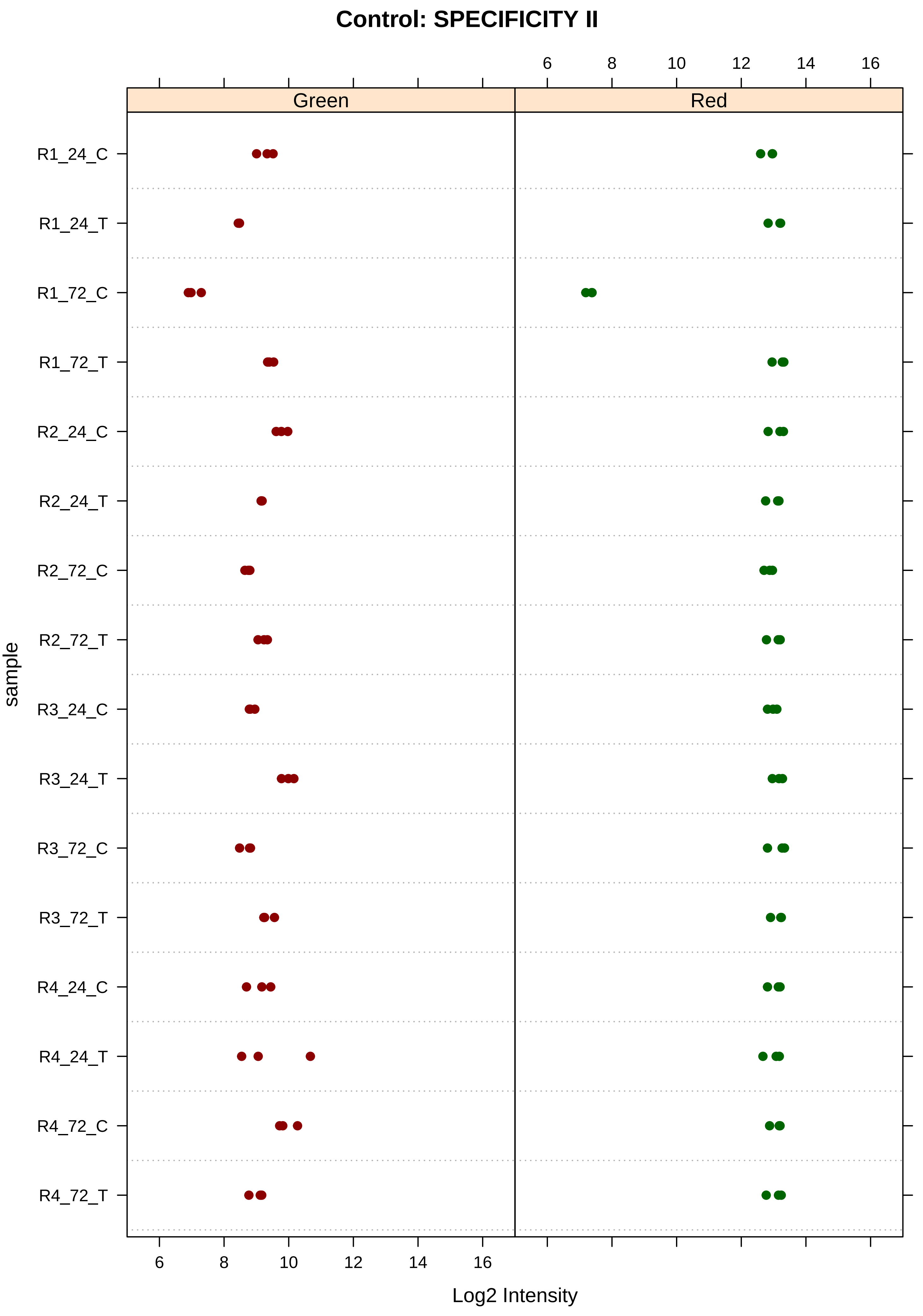
Control: SPECIFICITY II
Green
Red
R1_24_C
R1_24_T
R1_72_C
R1_72_T
R2_24_C
R2_24_T
R2_72_C
R2_72_T
R3_24_C
R3_24_T
R3_72_C
R3_72_T
R4_24_C
R4_24_T
R4_72_C
R4_72_T
sample
6
8
10
12
14
16
Log2 Intensity

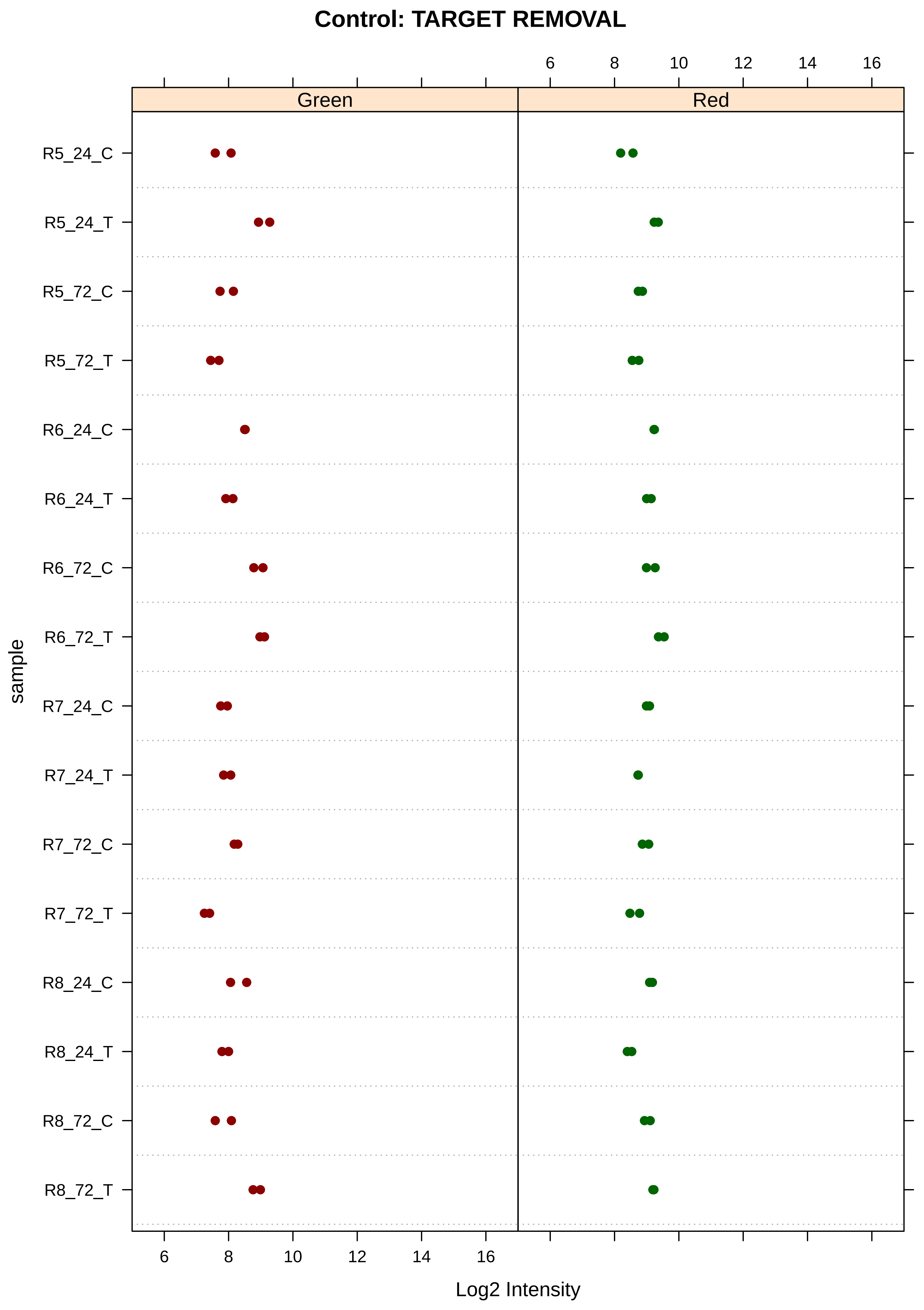

Control: TARGET REMOVAL
Green
Red
sample
R5_24_C
R5_24_T
R5_72_C
R5_72_T
R6_24_C
R6_24_T
R6_72_C
R6_72_T
R7_24_C
R7_24_T
R7_72_C
R7_72_T
R8_24_C
R8_24_T
R8_72_C
R8_72_T
6
8
10
12
14
16
Log2 Intensity

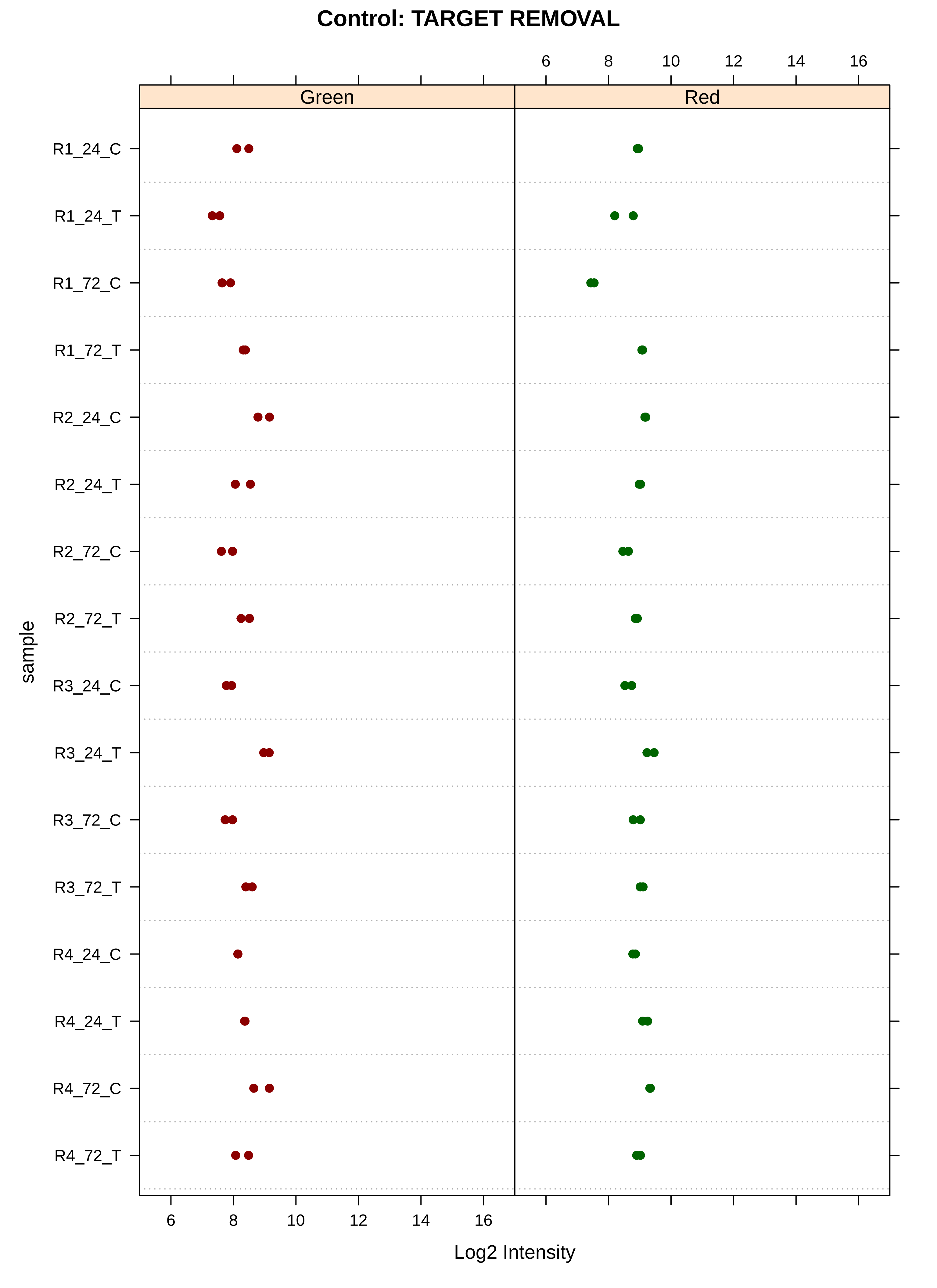
Control: TARGET REMOVAL
Green
Red
sample
R1_24_C
R1_24_T
R1_72_C
R1_72_T
R2_24_C
R2_24_T
R2_72_C
R2_72_T
R3_24_C
R3_24_T
R3_72_C
R3_72_T
R4_24_C
R4_24_T
R4_72_C
R4_72_T
6
8
10
12
14
16
Log2 Intensity
